# Abortion Surveillance — United States, 2015

**DOI:** 10.15585/mmwr.ss6713a1

**Published:** 2018-11-23

**Authors:** Tara C. Jatlaoui, Maegan E. Boutot, Michele G. Mandel, Maura K. Whiteman, Angeline Ti, Emily Petersen, Karen Pazol

**Affiliations:** 1 Division of Reproductive Health, National Center for Chronic Disease Prevention and Health Promotion, CDC; 2 Oak Ridge Institute for Science and Education (ORISE) Fellow

## Abstract

**Problem/Condition:**

Since 1969, CDC has conducted abortion surveillance to document the number and characteristics of women obtaining legal induced abortions in the United States.

**Period Covered:**

2015.

**Description of System:**

Each year, CDC requests abortion data from the central health agencies of 52 reporting areas (the 50 states, the District of Columbia, and New York City). The reporting areas provide this information voluntarily. For 2015, data were received from 49 reporting areas. Abortion data provided by these 49 reporting areas for each year during 2006–2015 were used in trend analyses. Census and natality data were used to calculate abortion rates (number of abortions per 1,000 women aged 15–44 years) and ratios (number of abortions per 1,000 live births), respectively.

**Results:**

A total of 638,169 abortions for 2015 were reported to CDC from 49 reporting areas. Among these 49 reporting areas, the abortion rate for 2015 was 11.8 abortions per 1,000 women aged 15–44 years, and the abortion ratio was 188 abortions per 1,000 live births. From 2014 to 2015, the total number of reported abortions decreased 2% (from 652,639), the abortion rate decreased 2% (from 12.1 abortions per 1,000 women aged 15–44 years), and the abortion ratio decreased 2% (from 192 abortions per 1,000 live births). From 2006 to 2015, the total number of reported abortions decreased 24% (from 842,855), the abortion rate decreased 26% (from 15.9 abortions per 1,000 women aged 15–44 years), and the abortion ratio decreased 19% (from 233 abortions per 1,000 live births). In 2015, all three measures reached their lowest level for the entire period of analysis (2006–2015).

In 2015 and throughout the period of analysis, women in their 20s accounted for the majority of abortions and had the highest abortion rates; women aged ≥30 years accounted for a smaller percentage of abortions and had lower abortion rates. In 2015, women aged 20–24 and 25–29 years accounted for 31.1% and 27.6% of all reported abortions, respectively, and had abortion rates of 19.9 and 17.9 abortions per 1,000 women aged 20–24 and 25–29 years, respectively. In contrast, women aged 30–34, 35–39, and ≥40 years accounted for 17.7%, 10.0%, and 3.5% of all reported abortions, respectively, and had abortion rates of 11.6, 7.0, and 2.5 abortions per 1,000 women aged 30–34, 35–39, and ≥40 years, respectively. From 2006 to 2015, the abortion rate decreased among women in all age groups.

In 2015, adolescents aged <15 and 15–19 years accounted for 0.3% and 9.8% of all reported abortions, respectively, and had abortion rates of 0.5 and 6.7 abortions per 1,000 adolescents aged <15 and 15–19 years, respectively. From 2006 to 2015, the percentage of abortions accounted for by adolescents aged 15–19 years decreased 41%, and their abortion rate decreased 54%. This decrease in abortion rate was greater than the decreases for women in any older age group.

In contrast to the percentage distribution of abortions and abortion rates by age, abortion ratios in 2015 and throughout the entire period of analysis were highest among adolescents and lowest among women aged 25–39 years. Abortion ratios decreased from 2006 to 2015 for women in all age groups.

In 2015, almost two thirds (65.4%) of abortions were performed at ≤8 weeks’ gestation, and nearly all (91.1%) were performed at ≤13 weeks’ gestation. Few abortions were performed between 14 and 20 weeks’ gestation (7.6%) or at ≥21 weeks’ gestation (1.3%). During 2006–2015 the percentage of all abortions performed at >13 weeks’ gestation remained consistently low (≤9.0%). Among abortions performed at ≤13 weeks’ gestation, a shift occurred toward earlier gestational ages, with the percentage performed at ≤6 weeks’ gestation increasing 11%.

In 2015, 24.6% of all abortions were performed by early medical abortion (a nonsurgical abortion at ≤8 weeks’ gestation), 64.3% were performed by surgical abortion at ≤13 weeks’ gestation, and 8.8% were performed by surgical abortion at >13 weeks’ gestation; all other methods were uncommon (≤2.2%). Among those that were eligible for early medical abortion on the basis of gestational age (i.e., performed at ≤8 weeks’ gestation), 35.8% were completed by this method.

In 2015, women with one or more previous live births accounted for 59.3% of abortions, and women with no previous live births accounted for 40.7%. Women with one or more previous induced abortions accounted for 43.6% of abortions, and women with no previous abortion accounted for 56.3%. Women with three or more previous births accounted for 14.2% of abortions, and women with three or more previous abortions accounted for 8.2% of abortions.

Deaths of women associated with complications from abortion for 2015 are being assessed as part of CDC’s Pregnancy Mortality Surveillance System. In 2014, the most recent year for which data were available, six women were identified to have died as a result of complications from legal induced abortion.

**Interpretation:**

Among the 49 areas that reported data every year during 2006–2015, decreases in the total number, rate, and ratio of reported abortions resulted in historic lows for the period of analysis for all three measures of abortion.

**Public Health Action:**

The data in this report can help program planners and policymakers identify groups of women with the highest rates of abortion. Unintended pregnancy is the major contributor to induced abortion. Increasing access to and use of effective contraception can reduce unintended pregnancies and further reduce the number of abortions performed in the United States.

## Introduction

This report summarizes abortion data for 2015 that were provided voluntarily to CDC by the central health agencies of 49 reporting areas (the District of Columbia [DC]; New York City; and 47 states, [excluding California, Maryland, and New Hampshire]). Data obtained every year during 2006–2015 from these same 49 reporting areas were used for trend analyses.

Since 1969, CDC has conducted abortion surveillance to document the number and characteristics of women obtaining legal induced abortions in the United States (*1*). After nationwide legalization of abortion in 1973, the total number, rate (number of abortions per 1,000 women aged 15–44 years), and ratio (number of abortions per 1,000 live births) of reported abortions increased rapidly, reaching the highest levels in the 1980s before decreasing at a slow yet steady pace (*2*–*4*). During 2006–2008, a break occurred in the previously sustained pattern of decrease (*5*–*8*), although this break has been followed in all subsequent years by even greater decreases (*9*–*16*). Nonetheless, throughout the years, the incidence of abortion has varied considerably across subpopulations and remains higher in certain demographic groups than others (*17*–*22*). Continued surveillance is needed to monitor changes in the incidence of abortion in the United States.

## Methods

### Description of the Surveillance System

Each year, CDC requests aggregated data from the central health agencies of 52 reporting areas (the 50 states, DC, and New York City) to document the number and characteristics of women obtaining legal induced abortions in the United States. This report contains data reported to CDC as of April 1, 2018. For the purpose of surveillance, a legal induced abortion* is defined as an intervention performed within the limits of state law by a licensed clinician (e.g., a physician, nurse-midwife, nurse practitioner, or physician assistant) that is intended to terminate a suspected or known intrauterine pregnancy.

In most states, collection of abortion data are facilitated by the legal requirement for hospitals, facilities, and physicians to report all abortions to a central health agency (*23*). These central health agencies then voluntarily report the abortion data they have collected through their independent surveillance systems (*24*). However, although reporting to CDC is voluntary, most reporting areas provide their abortion numbers.

Although CDC obtains abortion numbers from most of the central health agencies, it receives only aggregate numbers and reporting is not complete in all areas, including in certain areas with reporting requirements (*24*). Moreover, the level of detail received on the characteristics of women obtaining abortions varies considerably from year to year and by reporting area (*15*). To encourage more uniform collection of these details, CDC has collaborated with the National Association for Public Health Statistics and Information Systems to develop reporting standards and provide technical guidance for vital statistics personnel who collect and summarize abortion data within the United States. However, because the collection and reporting of abortion data are not federally mandated, many reporting areas have developed their own data collection forms, and therefore do not collect or provide all the information or level of detail included in this report.

### Variables and Categorization of Data

Each year, CDC sends suggested templates to the central health agencies for compilation of abortion data in aggregate. Aggregate abortion numbers, without individual-level records, are requested for the following variables:

- Maternal age in years (<15, 15–19 by individual year, 20–24, 25–29, 30–34, 35–39, or ≥40)
- Gestational age in completed weeks at the time of abortion (≤6, 7–20 by individual week, or ≥21)
- Race (black, white, or other [including Asian, Pacific Islander, other races, and multiple races]), ethnicity (Hispanic or non-Hispanic), and race by ethnicity
- Method type (surgical abortion,^†^ intrauterine instillation, medical [nonsurgical] abortion, or hysterectomy/hysterotomy)
- Marital status (married [including currently married or separated] or unmarried [including never married, widowed, or divorced])
- Number of previous live births (0, 1, 2, 3, or ≥4)
- Number of previous abortions (0, 1, 2, or ≥3)
- Maternal residence (the state, reporting area, territory, or foreign country in which the woman obtaining the abortion lived; or, if additional details are unavailable, in-reporting area versus out-of-reporting area)

In addition, templates provided by CDC request that aggregate numbers for certain variables be cross-tabulated by a second variable. These cross-tabulations include gestational age (separately by maternal age, by method type, by race, by ethnicity, and by race/ethnicity) and maternal age and marital status (separately by race, by ethnicity, and by race/ethnicity).

Beginning with 2014 data, instead of reporting clinician’s estimates of gestational age or estimates of gestational age on the basis of last menstrual period, certain areas reported “probable postfertilization age” and “clinician’s estimate of gestation based on date of conception” to CDC. To make data reported as postfertilization age consistent with gestational age data collection practices recommended by the CDC’s National Center for Health Statistics (*25*), 2 weeks were added to probable postfertilization age. This method was used to account for time after last menstrual period until ovulation in a standard 28-day cycle, because fertilization occurs around the time of ovulation (*26*). No modifications were made to data reported as clinician’s estimate of gestation based on date of conception.

In this report, medical and surgical abortions are further categorized by gestational age. Early medical abortion is defined as the administration of medication or medications (typically mifepristone followed by misoprostol) to induce an abortion at ≤8 completed weeks’ gestation*^§^*; medical abortion at >8 completed weeks’ gestation is defined as the administration of medication or medications (typically serial vaginal prostaglandins, sometimes after mifepristone) to induce an abortion at >8 weeks’ gestation. For surgical abortions, abortions are categorized as having been performed at ≤13 weeks’ gestation or at >13 weeks’ gestation because of differences in technique used generally before and after 13 weeks (*28*). Finally, because intrauterine instillations cannot be performed early in gestation, abortions reported to have been performed by intrauterine instillation at ≤12 weeks’ gestation are excluded from calculation of the percentage of abortions by known method type.^¶^

### Measures of Abortion

Four measures of abortion are presented in this report: 1) the number of abortions in a given population, 2) the percentage of abortions obtained by women in a given population, 3) the abortion rate (number of abortions per 1,000 women aged 15–44 years or other specific group within a given population), and 4) the abortion ratio (number of abortions per 1,000 live births within a given population). Although total numbers and percentages are useful for determining how many women have obtained an abortion, abortion rates adjust for differences in population size and reflect how likely abortion is among women in particular groups. Abortion ratios measure the relative number of pregnancies in a population that end in abortion compared with live birth. Abortion ratios are influenced both by the proportion of pregnancies in a population that are unintended and the proportion of unintended pregnancies that end in abortion. Abortion ratios also are influenced by the proportion of intended pregnancies that end in abortion; however, intended pregnancies account for a very limited percentage of abortions (<5%) (*31*).

U.S. Census Bureau estimates of the resident female population of the United States were used as the denominator for calculating abortion rates (*32*–*41*). Overall abortion rates were calculated from the population of women aged 15–44 years living in the reporting areas that provided data. For adolescents aged <15 years, abortion rates were determined on the basis of the number of adolescents aged 13–14 years; similarly, for women aged ≥40 years, abortion rates were determined on the basis of the number of women aged 40–44 years. For the calculation of abortion ratios, live birth data were obtained from CDC natality files and included births to women of all ages living in the reporting areas that provided abortion data (*42*).

### Data Presentation and Analysis

This report provides state-specific and overall abortion numbers, rates, and ratios for the 49 areas that reported to CDC for 2015 (excludes California, Maryland, and New Hampshire). In addition, this report describes the characteristics of women who obtained abortions in 2015. Because the completeness of reporting on the characteristics of women varies by year and by variable, this report only describes the characteristics of women obtaining abortions in areas that met reporting standards (i.e., reported at least 20 abortions overall, provided data categorized in accordance with surveillance variables, and had <15% unknown values for a given characteristic). Abortion rates and ratios have been omitted for reporting areas with <20 abortions because results are considered unstable (*43*). Cells with a value in the range of 1–4 or cells that would allow for calculation of these values have been suppressed to maintain confidentiality.

Although most of the data are presented by the reporting area in which the abortions were performed, 48 reporting areas in 2015 also provided the number of abortions by maternal residence.** However, one area only reported abortions for its own residents (DC). Two other areas (Illinois and Wisconsin) reported abortions for in-state and out-of-state residents but did not report certain characteristics for out-of-state residents. Three other reporting areas (Iowa, Massachusetts, and New Mexico) provided only the total number of abortions for out-of-state residents without specifying individual states or areas of residence from which these women came. As a result, abortion statistics in this report by area of residence should be interpreted with caution and might underestimate the incidence of abortion, especially for reporting areas from which multiple women travel to other states to obtain abortion services.

To evaluate overall trends in the number, rate, and ratio of reported abortions, annual data are presented for the 49 areas that reported every year during 2006–2015. Linear regression analysis was used to assess the overall rate of change among these areas during the entire 10-year period of analysis (2006–2015) and during the first and second halves of the period of analysis (2006–2010 and 2011–2015). The percentage change in abortion measures from the most recent past year (2014 to 2015) and from the beginning to the end of the 10-year period of analysis (2006 to 2015) also were calculated for these same 49 areas. Consistent with previous reports, key findings are highlighted to provide observed changes over time and differences between groups. However, comparisons do not infer statistical significance, and lack of comment regarding the difference between values does not imply that no statistically significant difference exists.

For the analysis of certain additional variables (i.e., abortions by maternal age and gestational age), annual data are presented for areas that met reporting standards every year during 2006–2015; the percentage change was calculated from the beginning to the end of the 10-year period of analysis (2006 to 2015), from the beginning to the end of the first and second halves of this period (2006 to 2010 and 2011 to 2015), and from the most recent past year (2014 to 2015). For other variables (i.e., race/ethnicity, method type, marital status, number of previous abortions, and number of previous live births), annual data are not presented; areas were included if they met reporting standards for the years needed for percentage change calculations. To evaluate trends in the use of different methods for performing an abortion, reporting areas were included only if they met reporting standards and if they specifically included medical abortion as a method on their reporting form. Trend analyses for race/ethnicity are limited to a 9-year span (2007–2015) because few reporting areas reported data on race by ethnicity (race/ethnicity) before 2007. Medical abortions performed at 9 completed weeks are also reported for 2011 to 2015. These data are reported to monitor any changes in clinical practice that might have occurred with the accumulation of evidence on the safety and effectiveness of medical abortion past 63 days of gestation (≤8 completed weeks) (*44*) and changes in professional practice guidelines published in 2013 and 2014 (*45*,*46*). Both of these events preceded the 2016 U.S. Food and Drug Administration (FDA) extension of the gestational age limit for the use of mifepristone for early medical abortion to 70 days (≤9 completed weeks) (*47*).

Some of the 49 areas that reported for 2015 are not included in certain trend analyses when data did not meet reporting standards. As a result, summary measures for comparisons over time might differ from the point estimates presented for all areas that reported for 2015.

### Abortion Mortality

CDC has reported data on abortion-related deaths periodically since information on abortion mortality first was included in the 1972 abortion surveillance report (*15*,*48*). An abortion-related death is defined as a death resulting from a direct complication of an abortion (legal or illegal), an indirect complication caused by a chain of events initiated by an abortion, or an aggravation of a preexisting condition by the physiologic or psychologic effects of abortion (*49*). All deaths determined to be related causally to induced abortion are classified as abortion related regardless of the time between the abortion and death. In addition, any pregnancy-related death in which the pregnancy outcome was induced abortion regardless of the causal relation between the abortion and the death is considered an abortion-related death. An abortion is defined as legal only if it is performed by a licensed clinician within the limits of state law.

Since 1987, CDC has monitored abortion-related deaths through its Pregnancy Mortality Surveillance System (*50*,*51*). Sources of data for abortion-related deaths have included state vital records; media reports, including computerized searches of full-text newspaper and other print media databases; and individual case reports by public health agencies, including maternal mortality review committees, health care providers and provider organizations, private citizens, and citizen groups. For each death that possibly is related to abortion, CDC requests clinical records and autopsy reports. Two medical epidemiologists independently review these reports to determine the cause of death and whether the death was abortion related. Discrepancies are discussed and resolved by consensus. Each death is categorized by abortion type as legal induced, illegal induced, spontaneous, or unknown type.

This report provides data from the Pregnancy Mortality Surveillance System on induced abortion-related deaths that occurred in 2014, the most recent year for which data are available. Data on induced abortion-related deaths that occurred during 1972–2013 already have been published (*15*), and possible abortion-related deaths that occurred during 2015–2018 are being assessed. During 1998–2014, abortion surveillance data reported to CDC cannot be used alone to calculate national case-fatality rates (number of legal induced abortion-related deaths per 100,000 reported legal induced abortions in the United States) because certain states^††^ did not report abortion data every year during this period. Thus, national legal induced abortion case-fatality rates were calculated with denominator data from a provider census for the total number of abortions performed in the United States (*16*). Because rates determined on the basis of a numerator of <20 deaths are highly variable (*43*), national legal induced abortion case-fatality rates were calculated for consecutive 5-year periods during 1973–2007 and for a consecutive 7-year period during 2008–2014.

## Results

### U.S. Totals

**FIGURE 1:**
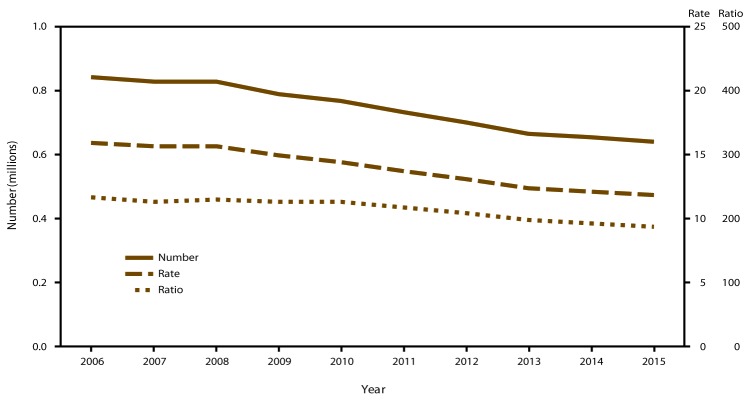
Number, rate,* and ratio^†^ of abortions performed, by year — selected reporting areas,^§^ United States, 2006–2015 * Number of abortions per 1,000 women aged 15–44 years. ^†^ Number of abortions per 1,000 live births. ^§^ Data are for 49 reporting areas; excludes California, Maryland, and New Hampshire.

Among the 49 reporting areas that provided data for 2015, a total of 638,169 abortions were reported. All 49 of these areas provided data every year during 2006–2015.^§§^ In 2015, these areas had an abortion rate of 11.8 abortions per 1,000 women aged 15–44 years and an abortion ratio of 188 abortions per 1,000 live births (Table 1). From 2014 to 2015, the total number of reported abortions decreased 2% (from 652,639 to 638,169), the abortion rate decreased 2% (from 12.1 to 11.8 abortions per 1,000 women aged 15–44 years), and the abortion ratio decreased 2% (from 192 to 188 abortions per 1,000 live births). From 2006 to 2015, the total number of reported abortions decreased 24% (from 842,855), the abortion rate decreased 26% (from 15.9 abortions per 1,000 women aged 15–44 years), and the abortion ratio decreased 19% (from 233 abortions per 1,000 live births) (Figure 1). Among these same 49 areas, the annual rate of decrease fitted from the regression analysis was greater during 2011–2015 than during 2006–2010 for all three measures of abortion. During 2011–2015, the number of reported abortions decreased by 23,087 abortions per year, the abortion rate decreased by 0.48 abortions per 1,000 women per year, and the abortion ratio decreased by 7.4 abortions per 1,000 live births per year. In contrast, during 2006–2010, the number of reported abortions decreased by 19,280 abortions per year, the abortion rate decreased by 0.37 abortions per 1,000 women per year, and the abortion ratio decreased by 1.7 abortions per 1,000 live births per year.

**TABLE 1:** Number, percentage, rate,* and ratio^†^ of reported abortions — selected reporting areas, United States, 2006–2015

Year	Selected reporting areas^§^	Continuously reporting areas^¶^		
	No.	No. (%)**	Rate	Ratio
2006	852,385^††^	842,855 (98.9)	15.9	233
2007	827,609	827,609 (100.0)	15.6	226
2008	825,564	825,564 (100.0)	15.6	229
2009	789,217^§§^	789,217 (100.0)	14.9	225
2010	765,651	765,651 (100.0)	14.4	225
2011	730,322	730,322 (100.0)	13.7	217
2012	699,202	699,202 (100.0)	13.1	208
2013	664,435	664,435 (100.0)	12.4	198
2014	652,639	652,639 (100.0)	12.1	192
2015	638,169	638,169 (100.0)	11.8	188

* Number of abortions per 1,000 women aged 15–44 years.

^†^ Number of abortions per 1,000 live births.

^§^ For each given year, excludes reporting areas that did not report that year’s abortion numbers to CDC: California (2006–2015), Maryland (2007–2015), and New Hampshire (2006–2015).

^¶^ For all years, excludes reporting areas that did not report abortion numbers every year during the period of analysis (2006–2015): California, Maryland, and New Hampshire.

** Abortions from areas that reported every year during 2006–2015 as a percentage of all reported abortions.

^††^ This number is greater than reported in the 2006 report because of numbers subsequently provided by Louisiana.

^§§^ This number is greater than reported in the 2009 report because of numbers subsequently provided by Delaware.

### Occurrence and Residence

Abortion numbers, rates, and ratios for 2015 have been calculated by reporting area of occurrence and the residence of the women who obtained the abortions (Table 2). By reporting area of occurrence, a considerable range existed in the abortion rate (from 2.8 abortions per 1,000 women aged 15–44 years in South Dakota to 23.1 abortions per 1,000 women in New York [city and state combined]) and the abortion ratio (from 36 abortions per 1,000 live births in South Dakota to 392 abortions per 1,000 live births in New York [city and state combined]).^¶¶^ Similarly, a considerable range existed by residence*** in the abortion rate (from 4.2 abortions per 1,000 women aged 15–44 years in South Dakota to 22.0 abortions per 1,000 women aged 15–44 years in New York [city and state combined]) and the abortion ratio (from 53 abortions per 1,000 live births in South Dakota to 374 abortions per 1,000 live births in New York [city and state combined]). Because of variation that occurred among reporting areas in the percentage of abortions obtained by out-of-state residents (from 0.3% in Hawaii to 49.0% in Kansas),^†††^ abortion rates and ratios calculated by maternal residence might provide a more accurate reflection of the state-specific distribution of women obtaining abortions. However, because states vary in the level of detail they collect on maternal residence, 12.7% of abortions were reported to CDC with unknown information on maternal residence.

**TABLE 2:** Number, rate,* and ratio^†^ of reported abortions, by reporting area of residence and occurrence and by percentage of abortions obtained by out-of-state residents — United States, 2015

State/Area	Residence			Occurrence			% obtained by out-of-state residents^§^
	No.	Rate	Ratio	No.	Rate	Ratio	
Alabama	6,618	6.9	111	5,899	6.2	99	13.1
Alaska	1,459	10.0	129	1,334	9.1	118	0.5
Arizona	12,644	9.6	148	12,655	9.6	148	1.4
Arkansas	3,805	6.6	98	3,771	6.5	97	18.6
California^¶^	—	—	—	—	—	—	—
Colorado	8,975	8.1	135	10,114	9.1	152	11.3
Connecticut	9,888	14.5	277	9,938	14.6	278	2.7
Delaware	2,889	16.0	259	2,785	15.4	249	15.0
District of Columbia**	1,424	7.9	149	1,267	7.0	132	—
Florida^††^	—	—	—	72,023	19.3	321	—
Georgia	26,835	12.7	204	31,009	14.6	236	14.5
Hawaii	2,042	7.6	111	2,026	7.6	110	0.3
Idaho	1,695	5.3	74	1,272	4.0	56	4.6
Illinois	35,237	13.7	223	39,856	15.5	252	8.5
Indiana	9,546	7.4	114	7,957	6.1	95	5.9
Iowa^§§^	3,676	6.2	93	3,989	6.8	101	16.3
Kansas	3,637	6.5	93	6,931	12.4	177	49.0
Kentucky	4,585	5.4	82	3,188	3.7	57	12.8
Louisiana	8,515	9.0	132	9,362	9.9	145	14.6
Maine	1,743	7.5	138	1,836	7.9	146	3.1
Maryland^¶^	—	—	—	—	—	—	—
Massachusetts^§§^	17,959	13.1	251	18,570	13.5	260	3.8
Michigan	26,283	14.0	232	27,151	14.4	240	4.2
Minnesota	9,234	8.8	132	9,861	9.4	141	9.8
Mississippi	4,699	7.8	122	2,613	4.4	68	5.1
Missouri	8,636	7.3	115	4,765	4.0	63	9.5
Montana	1,433	7.7	114	1,611	8.6	128	13.3
Nebraska	1,893	5.2	71	2,004	5.5	75	11.4
Nevada	6,760	11.8	186	7,116	12.4	196	5.5
New Hampshire^¶^	—	—	—	—	—	—	—
New Jersey^¶¶^	23,224	13.5	225	22,991	13.4	223	5.5
New Mexico^§§^	3,496	8.8	135	4,669	11.8	181	27.2
New York	88,762	22.0	374	93,096	23.1	392	5.1
New York City	NA	NA	NA	63,646	32.8	544	NA
New York State	NA	NA	NA	29,450	14.1	245	NA
North Carolina	23,066	11.6	191	27,631	13.9	229	17.5
North Dakota	976	6.6	86	1,166	7.9	103	29.6
Ohio	21,215	9.6	152	20,976	9.5	151	5.8
Oklahoma	4,813	6.3	91	4,709	6.1	89	8.0
Oregon	7,847	10.0	172	8,610	10.9	189	11.2
Pennsylvania	32,025	13.3	227	31,818	13.3	226	5.0
Rhode Island	2,348	11.2	214	2,649	12.6	241	14.7
South Carolina	11,032	11.6	190	5,778	6.1	99	5.9
South Dakota	659	4.2	53	444	2.8	36	13.3
Tennessee	10,361	8.0	127	11,411	8.8	140	20.3
Texas	54,194	9.4	134	53,940	9.4	134	1.8
Utah	3,123	4.8	62	3,176	4.9	63	6.0
Vermont	1,121	9.7	190	1,265	10.9	214	12.4
Virginia	18,501	11.0	179	18,663	11.1	181	5.2
Washington	17,230	12.2	194	17,098	12.1	192	4.5
West Virginia	1,736	5.2	88	1,516	4.5	77	12.7
Wisconsin	6,731	6.2	100	5,660	5.2	84	3.5
Wyoming	599	5.4	77	—***	—^†††^	—^†††^	—^†††^
Canada	83	NA	NA	NA	NA	NA	NA
Mexico	256	NA	NA	NA	NA	NA	NA
Other country or territory	237	NA	NA	NA	NA	NA	NA
**Total known**	**557,304**	**NA**	**NA**	**NA**	**NA**	**NA**	**NA**
**Percentage reported by known residence**	**87.3**	**NA**	**NA**	**NA**	**NA**	**NA**	**NA**
**Total unknown residence**	**80,865**	**NA**	**NA**	**NA**	**NA**	**NA**	**NA**
Out of state, exact residence not stated	5,340	NA	NA	NA	NA	NA	NA
No information on residence provided	75,525	NA	NA	NA	NA	NA	NA
**Percentage reported by unknown residence**	**12.7**	**NA**	**NA**	**NA**	**NA**	**NA**	**NA**
**Total**	**638,169**	**NA**	**NA**	**NA**	**NA**	**NA**	**NA**

**Abbreviation**: NA = not applicable.

* Number of abortions per 1,000 women aged 15–44 years.

^†^ Number of abortions per 1,000 live births.

^§^ Additional details on the state in which abortions were provided, cross-tabulated by the state of maternal residence, are at http://www.cdc.gov/reproductivehealth/data_stats/Abortion.htm.

^¶^ Reporting area did not report; because numbers for this area are available only from other reporting areas where residents obtained abortions, meaningful statistics cannot be reported.

** Because reporting is not mandatory, a complete count of the number of abortions performed in the District of Columbia could not be obtained and were only reported for area residents.

^††^ Reported by occurrence only; because abortion numbers by residence for Florida are available only from other states where residents obtained abortions, meaningful statistics cannot be reported.

^§§^ Reporting area reported abortion numbers for both in-state and out-of-state residents; for out-of-state residents, the state or area of residence was not provided.

^¶¶^ Data from hospitals and licensed ambulatory care facilities only; because reporting is not mandatory for private physicians and women's centers, a complete count of the number of abortions performed in New Jersey could not be obtained.

*** Total abortion number <20.

^†††^ Abortion rates and ratios and percentage of abortions obtained by out-of-state residents were not calculated for Wyoming because results based on a small number of abortions are unstable.

### Maternal Age

**FIGURE 2:**
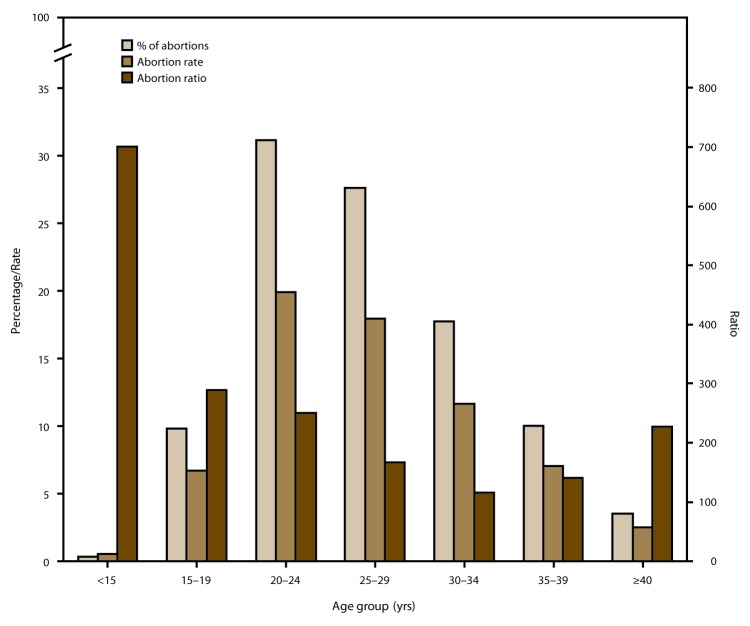
Percentage of total abortions, abortion rate,* and abortion ratio,^†^ by age group of women who obtained a legal abortion — selected reporting areas,^§^ United States, 2015 * Number of abortions per 1,000 women aged 15–44 years. ^†^ Number of abortions per 1,000 live births. ^§^ Data are for 47 areas; excludes five areas (California, Florida, Maryland, New Hampshire, and Wyoming) that did not report, did not report by age, or did not meet reporting standards.

Among the 47 areas that reported by maternal age for 2015, women in their 20s accounted for the majority (58.7%) of abortions and had the highest abortion rates (19.9 and 17.9 abortions per 1,000 women aged 20–24 and 25–29 years, respectively) (Figure 2) (Table 3). Women in the youngest (<15 years) and oldest (≥40 years) age groups accounted for the smallest percentages of abortions (0.3% and 3.5%, respectively) and had the lowest abortion rates (0.5 and 2.5 abortions per 1,000 women aged <15 and ≥40 years, respectively). Among the 44 reporting areas that provided data by maternal age every year during 2006–2015, this pattern across age groups was stable, with the majority of abortions and the highest abortion rates occurring among women aged 20–29 years and the lowest percentages of abortions and abortion rates occurring among women in the youngest and oldest age groups (Table 4). From 2006 to 2015, abortion rates decreased among all age groups, although the decreases for adolescents (58% and 54% for adolescents aged <15 and 15–19 years, respectively) were greater than the decreases for women in all older age groups, with decreases for women aged ≥20 years ranging from 4% among women aged ≥40 years to 33% among women aged 20–24 years. Decreases in the abortion rate for all age groups, except women aged 25–29 years, were greater from 2011 to 2015 than from 2006 to 2010, and the rates for all age groups either remained the same or decreased from 2014 to 2015.

**TABLE 3:** Reported abortions, by known age group and reporting area of occurrence — selected reporting areas,* United States, 2015

State/Area	Age group (yrs)							Total abortions reported by known age
	<15	15–19	20–24	25–29	30–34	35–39	≥40	
	No. (%)^†^	No. (%)	No. (%)	No. (%)	No. (%)	No. (%)	No. (%)	No. (% of all reported abortions)^§^
**Alabama**	23 (0.4)	643 (10.9)	2,085 (35.3)	1,582 (26.8)	953 (16.2)	478 (8.1)	135 (2.3)	**5,899 (100.0)**
Alaska	—^¶^	152 (11.4)	445 (33.4)	361 (27.1)	206 (15.4)	121 (9.1)	—	**1,334 (100.0)**
Arizona	25 (0.2)	1,177 (9.3)	3,948 (31.2)	3,416 (27.0)	2,228 (17.6)	1,344 (10.6)	513 (4.1)	**12,651 (100.0)**
Arkansas	16 (0.4)	391 (10.4)	1,214 (32.2)	997 (26.4)	693 (18.4)	344 (9.1)	116 (3.1)	**3,771 (100.0)**
Colorado	27 (0.3)	1,019 (10.1)	3,196 (31.8)	2,735 (27.2)	1,712 (17.0)	993 (9.9)	372 (3.7)	**10,054 (99.4)**
Connecticut	21 (0.2)	992 (10.2)	2,931 (30.1)	2,735 (28.1)	1,768 (18.2)	960 (9.9)	321 (3.3)	**9,728 (97.9)**
Delaware	5 (0.2)	328 (11.8)	892 (32.0)	747 (26.8)	466 (16.7)	264 (9.5)	83 (3.0)	**2,785 (100.0)**
District of Columbia**	—	113 (8.9)	406 (32.0)	390 (30.8)	227 (17.9)	99 (7.8)	—	**1,267 (100.0)**
Georgia	86 (0.3)	2,783 (9.0)	9,638 (31.1)	8,650 (27.9)	5,558 (17.9)	3,178 (10.2)	1,116 (3.6)	**31,009 (100.0)**
Hawaii	5 (0.2)	215 (10.7)	580 (28.8)	529 (26.3)	356 (17.7)	235 (11.7)	94 (4.7)	**2,014 (99.4)**
Idaho	—	165 (13.0)	433 (34.0)	274 (21.5)	202 (15.9)	144 (11.3)	—	**1,272 (100.0)**
Illinois^††^	82 (0.2)	3,415 (9.9)	10,794 (31.4)	9,658 (28.1)	5,928 (17.2)	3,403 (9.9)	1,113 (3.2)	**34,393 (99.7)**
Indiana	25 (0.3)	821 (10.3)	2,686 (33.8)	2,111 (26.5)	1,279 (16.1)	780 (9.8)	255 (3.2)	**7,957 (100.0)**
Iowa	13 (0.3)	445 (11.2)	1,316 (33.0)	1,028 (25.8)	700 (17.6)	360 (9.0)	120 (3.0)	**3,982 (99.8)**
Kansas	13 (0.2)	657 (9.5)	2,190 (31.6)	1,829 (26.4)	1,281 (18.5)	735 (10.6)	226 (3.3)	**6,931 (100.0)**
Kentucky	16 (0.5)	337 (10.6)	1,037 (32.5)	851 (26.7)	521 (16.3)	288 (9.0)	138 (4.3)	**3,188 (100.0)**
Louisiana	37 (0.4)	828 (8.8)	2,928 (31.3)	2,758 (29.5)	1,662 (17.8)	884 (9.4)	264 (2.8)	**9,361 (100.0)**
Maine	5 (0.3)	206 (11.2)	582 (31.7)	478 (26.0)	292 (15.9)	206 (11.2)	66 (3.6)	**1,835 (99.9)**
Massachusetts	27 (0.1)	1,547 (8.3)	5,492 (29.6)	5,342 (28.8)	3,416 (18.4)	1,964 (10.6)	777 (4.2)	**18,565 (100.0)**
Michigan	84 (0.3)	2,579 (9.5)	8,970 (33.1)	7,610 (28.1)	4,388 (16.2)	2,595 (9.6)	848 (3.1)	**27,074 (99.7)**
Minnesota	18 (0.2)	854 (8.7)	2,939 (29.8)	2,690 (27.3)	1,912 (19.4)	1,107 (11.2)	341 (3.5)	**9,861 (100.0)**
Mississippi	13 (0.5)	258 (9.9)	875 (33.5)	748 (28.6)	450 (17.2)	210 (8.0)	59 (2.3)	**2,613 (100.0)**
Missouri	14 (0.3)	480 (10.1)	1,647 (34.6)	1,278 (26.8)	779 (16.4)	413 (8.7)	153 (3.2)	**4,764 (100.0)**
Montana	—	201 (12.5)	515 (32.0)	419 (26.0)	269 (16.7)	145 (9.0)	—	**1,610 (99.9)**
Nebraska	7 (0.3)	194 (9.7)	576 (28.7)	578 (28.8)	360 (18.0)	223 (11.1)	66 (3.3)	**2,004 (100.0)**
Nevada	12 (0.2)	636 (9.2)	1,974 (28.6)	1,906 (27.6)	1,350 (19.6)	713 (10.3)	313 (4.5)	**6,904 (97.0)**
New Jersey^§§^	53 (0.2)	2,234 (9.7)	6,649 (28.9)	6,569 (28.6)	4,124 (18.0)	2,411 (10.5)	931 (4.1)	**22,971 (99.9)**
New Mexico	24 (0.5)	614 (13.7)	1,425 (31.9)	1,112 (24.9)	763 (17.1)	370 (8.3)	158 (3.5)	**4,466 (95.7)**
New York	241 (0.3)	9,303 (10.0)	27,481 (29.6)	25,365 (27.3)	17,110 (18.4)	9,785 (10.5)	3,685 (4.0)	**92,970 (99.9)**
New York City	153 (0.2)	5,796 (9.1)	18,148 (28.5)	17,626 (27.7)	12,045 (18.9)	7,156 (11.2)	2,720 (4.3)	**63,644 (100.0)**
New York State	88 (0.3)	3,507 (12.0)	9,333 (31.8)	7,739 (26.4)	5,065 (17.3)	2,629 (9.0)	965 (3.3)	**29,326 (99.6)**
North Carolina	70 (0.3)	2,486 (9.5)	8,388 (32.1)	7,277 (27.8)	4,451 (17.0)	2,567 (9.8)	892 (3.4)	**26,131 (94.6)**
North Dakota	6 (0.5)	107 (9.2)	395 (33.9)	290 (24.9)	211 (18.1)	121 (10.4)	36 (3.1)	**1,166 (100.0)**
Ohio	73 (0.3)	2,114 (10.1)	6,809 (32.5)	5,975 (28.5)	3,441 (16.4)	1,909 (9.1)	655 (3.1)	**20,976 (100.0)**
Oklahoma	10 (0.2)	515 (10.9)	1,554 (33.0)	1,215 (25.8)	837 (17.8)	423 (9.0)	154 (3.3)	**4,708 (100.0)**
Oregon	21 (0.2)	901 (10.5)	2,622 (30.5)	2,297 (26.7)	1,551 (18.0)	871 (10.1)	344 (4.0)	**8,607 (100.0)**
Pennsylvania	86 (0.3)	3,028 (9.5)	10,339 (32.5)	9,119 (28.7)	5,303 (16.7)	2,951 (9.3)	992 (3.1)	**31,818 (100.0)**
Rhode Island	8 (0.3)	241 (9.1)	837 (31.7)	727 (27.5)	434 (16.4)	279 (10.6)	114 (4.3)	**2,640 (99.7)**
South Carolina	12 (0.2)	602 (10.4)	1,884 (32.6)	1,566 (27.1)	962 (16.6)	550 (9.5)	202 (3.5)	**5,778 (100.0)**
South Dakota	—	41 (9.2)	145 (32.7)	112 (25.2)	85 (19.1)	46 (10.4)	—	**444 (100.0)**
Tennessee	34 (0.3)	1,075 (9.5)	3,627 (32.2)	3,159 (28.0)	1,988 (17.6)	1,049 (9.3)	334 (3.0)	**11,266 (98.7)**
Texas	151 (0.3)	5,001 (9.3)	16,738 (31.0)	15,016 (27.8)	9,592 (17.8)	5,490 (10.2)	1,946 (3.6)	**53,934 (100.0)**
Utah	0 (0.0)	363 (11.5)	1,012 (32.1)	808 (25.6)	520 (16.5)	320 (10.1)	131 (4.2)	**3,154 (99.3)**
Vermont	—	127 (10.2)	383 (30.8)	335 (27.0)	240 (19.3)	116 (9.3)	—	**1,243 (98.3)**
Virginia	38 (0.2)	1,558 (8.4)	5,686 (30.6)	5,086 (27.4)	3,554 (19.1)	1,979 (10.7)	677 (3.6)	**18,578 (99.5)**
Washington	36 (0.2)	1,905 (11.1)	5,061 (29.6)	4,469 (26.1)	3,117 (18.2)	1,813 (10.6)	692 (4.0)	**17,093 (100.0)**
West Virginia	6 (0.4)	145 (9.6)	478 (31.6)	394 (26.1)	304 (20.1)	145 (9.6)	40 (2.6)	**1,512 (99.7)**
Wisconsin^††^	13 (0.2)	623 (11.4)	1,813 (33.2)	1,403 (25.7)	955 (17.5)	482 (8.8)	172 (3.1)	**5,461 (100.0)**
**Total**	**1,471 (0.3)**	**54,419 (9.8)**	**173,615 (31.1)**	**153,994 (27.6)**	**98,498 (17.7)**	**55,863 (10.0)**	**19,882 (3.5)**	**557,742** **(99.5)**^¶¶^
Abortion rate***	**0.5**	**6.7**	**19.9**	**17.9**	**11.6**	**7.0**	**2.5**	**11.2**
**Abortion ratio** ^†††^	**701**	**289**	**250**	**167**	**115**	**140**	**227**	**177**

* Data from 47 reporting areas; excludes five reporting areas (California, Florida, Maryland, New Hampshire, and Wyoming) that did not report, did not report by age, or did not meet reporting standards.

^†^ Percentages for the individual component categories might not add to 100 because of rounding.

^§^ Percentage is calculated as the number of abortions reported by known age divided by the sum of abortions reported by known and unknown age.

^¶^ Cells with a value in the range of 1–4 or cells that would allow for calculation of these small values have been suppressed.

** Because reporting is not mandatory, information could not be obtained for all abortions performed in the District of Columbia.

^††^ Includes residents only.

^§§^ Data from hospitals and licensed ambulatory care facilities only; because reporting is not mandatory for private physicians and women's centers, information could not be obtained for all abortions performed in New Jersey.

^¶¶^ Percentage based on a total of 560,589 abortions reported among the areas that met reporting standards for age.

*** Number of abortions obtained by women in a given age group per 1,000 women in that same age group. Women aged 13–14 years were used as the denominator for the group of women aged <15 years, and women aged 40–44 years were used as the denominator for the group of women aged ≥40 years. Women aged 15–44 years were used as the denominator for the overall rate. For each reporting area, abortions for women of unknown age were distributed according to the distribution of abortions among women of known age for that area.

^†††^ Number of abortions obtained by women in a given age group per 1,000 live births to women in that same age group. For each reporting area, abortions for women of unknown age were distributed according to the distribution of abortions among women of known age for that area.

**TABLE 4:** Reported abortions, by known age group and year — selected reporting areas,* United States, 2006–2015

Year											% change			
Age group (yrs)	2006	2007	2008	2009	2010	2011	2012	2013	2014	2015	2006 to 2010	2011 to 2015	2014 to 2015	2006 to 2015
**% of abortions**														
<15	0.5	0.5	0.5	0.5	0.5	0.4	0.4	0.3	0.3	0.3	0.0	-25.0	0.0	**-40.0**
15–19	16.5	16.5	16.1	15.5	14.6	13.5	12.2	11.4	10.4	9.8	-11.5	-27.4	-5.8	**-40.6**
20–24	32.7	32.7	32.7	32.7	32.9	32.9	32.8	32.7	32.1	31.1	0.6	-5.5	-3.1	**-4.9**
25–29	24.1	24.2	24.4	24.4	24.5	24.9	25.4	25.9	26.8	27.6	1.7	10.8	3.0	**14.5**
30–34	14.2	14.1	14.3	14.8	15.3	15.8	16.4	16.8	17.2	17.7	7.7	12.0	2.9	**24.6**
35–39	8.8	8.8	8.8	8.8	8.9	8.9	9.1	9.2	9.7	10.0	1.1	12.4	3.1	**13.6**
≥40	3.1	3.2	3.1	3.3	3.4	3.6	3.7	3.6	3.6	3.6	9.7	0.0	0.0	**16.1**
**Abortion rate^†^**														
<15	1.2	1.2	1.2	1.1	1.0	0.9	0.8	0.6	0.5	0.5	-16.7	-44.4	0.0	**-58.3**
15–19	14.5	14.1	13.8	12.8	11.7	10.5	9.2	8.2	7.3	6.7	-19.3	-36.2	-8.2	**-53.8**
20–24	29.7	29.2	29.3	27.7	26.8	25.0	23.3	21.9	20.9	19.9	-9.8	-20.4	-4.8	**-33.0**
25–29	22.5	21.8	21.8	20.7	20.2	19.4	18.9	18.2	18.1	17.9	-10.2	-7.7	-1.1	**-20.4**
30–34	13.8	13.6	13.8	13.4	13.2	12.7	12.4	11.8	11.7	11.7	-4.3	-7.9	0.0	**-15.2**
35–39	7.9	7.8	7.8	7.6	7.6	7.5	7.3	7.0	7.1	7.0	-3.8	-6.7	-1.4	**-11.4**
≥40	2.6	2.6	2.7	2.8	2.8	2.8	2.8	2.5	2.5	2.5	7.7	-10.7	0.0	**-3.8**
**Abortion ratio^§^**														
<15	747	770	804	832	848	839	804	791	745	700	13.5	-16.6	-6.0	**-6.3**
15–19	349	336	337	328	332	325	304	299	291	289	-4.9	-11.1	-0.7	**-17.2**
20–24	278	274	283	281	290	284	272	262	256	250	4.3	-12.0	-2.3	**-10.1**
25–29	189	183	186	183	184	178	174	168	166	167	-2.6	-6.2	0.6	**-11.6**
30–34	142	137	140	138	138	132	128	121	116	115	-2.8	-12.9	-0.9	**-19.0**
35–39	173	170	174	172	171	165	158	147	145	140	-1.2	-15.2	-3.4	**-19.1**
≥40	283	278	271	275	273	275	269	245	239	228	-3.5	-17.1	-4.6	**-19.4**
**Total (no.)**	**732,654**	**722,831**	**726,839**	**695,952**	**675,732**	**643,628**	**614,570**	**582,260**	**569,100**	**556,221**	—	—	—	**—**

* Data from 44 reporting areas; by year, these reporting areas represent 98%–99% of all abortions reported to CDC by age during 2006–2015. Excludes eight reporting areas (California, District of Columbia, Florida, Maine, Maryland, New Hampshire, Vermont, and Wyoming) that did not report, did not report by age, or did not meet reporting standards for ≥1 year.

^†^ Number of abortions obtained by women in a given age group per 1,000 women in that same age group. Women aged 13–14 years were used as the denominator for the group of women aged <15 years, and women aged 40–44 years were used as the denominator for the group of women aged ≥40 years. Women aged 15–44 years were used as the denominator for the overall rate. For each reporting area, abortions for women of unknown age were distributed according to the distribution of abortions among women of known age for that area.

^§^ Number of abortions obtained by women in a given age group per 1,000 live births to women in that same age group. For each reporting area, abortions for women of unknown age were distributed according to the distribution of abortions among women of known age for that area.

In contrast to the percentage of abortions and abortion rates, abortion ratios in 2015 were highest among adolescents aged ≤19 years and lowest among women aged 25–39 years (Figure 2) (Table 3). Among the 44 reporting areas that provided data by maternal age for every year during 2006–2015, abortion ratios decreased among women in all age groups. The abortion ratio decreased for all age groups from 2011 to 2015; from 2006 to 2010, it decreased for women in all age groups, except for those aged <15 and 20–24 years. In addition, for every age group with declines for both periods, the declines that occurred from 2011 to 2015 exceeded the declines from 2006 to 2010. Declines occurred for all age groups from 2014 to 2015 with the exception of women aged 25–29 years (Table 4).

### Adolescents

Among the 45 areas that reported maternal age by individual year among adolescents for 2015, adolescents aged 18–19 years accounted for the majority (67.8%) of adolescent abortions and had the highest adolescent abortion rates (9.6 and 13.2 abortions per 1,000 adolescents aged 18 and 19 years, respectively). Adolescents aged <15 years accounted for the smallest percentage of adolescent abortions (2.7%) and had the lowest adolescent abortion rate (0.5 abortions per 1,000 adolescents aged 13–14 years) (Table 5). Among the 40 reporting areas that provided maternal age data for adolescents for each individual year of reporting during 2006–2015, the percentage of abortions accounted for by adolescents aged 18–19 years increased, whereas the percentage of abortions accounted for by adolescents aged <18 years decreased (Table 6). For adolescents of all ages, large decreases in abortion rates occurred from 2006 to 2015 (48%–64%) and were greater from 2011 to 2015 than from 2006 to 2010. Decreases continued among all adolescents aged ≥15 years from 2014 to 2015.

**TABLE 5:** Reported abortions among adolescents, by known age and reporting area of occurrence — selected reporting areas,* United States, 2015

Age (yrs)							
State/Area	<15	15	16	17	18	19	Total no.
	No. (%)^†^	No. (%)	No. (%)	No. (%)	No. (%)	No. (%)	
Alabama	23 (3.5)	42 (6.3)	55 (8.3)	85 (12.8)	178 (26.7)	283 (42.5)	**666**
Alaska	—^§^	—	13 (8.3)	19 (12.2)	47 (30.1)	63 (40.4)	**156**
Arizona	25 (2.1)	50 (4.2)	85 (7.1)	153 (12.7)	358 (29.8)	531 (44.2)	**1,202**
Arkansas	16 (3.9)	28 (6.9)	40 (9.8)	57 (14.0)	113 (27.8)	153 (37.6)	**407**
Colorado	27 (2.6)	53 (5.1)	120 (11.5)	141 (13.5)	281 (26.9)	424 (40.5)	**1,046**
Connecticut	21 (2.1)	40 (3.9)	94 (9.3)	205 (20.2)	262 (25.9)	391 (38.6)	**1,013**
Delaware	5 (1.5)	23 (6.9)	26 (7.8)	67 (20.1)	96 (28.8)	116 (34.8)	**333**
District of Columbia^¶,^**	—	—	10 (8.6)	23 (19.8)	29 (25.0)	46 (39.7)	**116**
Georgia	86 (3.0)	174 (6.1)	284 (9.9)	411 (14.3)	782 (27.3)	1,132 (39.5)	**2,869**
Hawaii	5 (2.3)	14 (6.4)	24 (10.9)	36 (16.4)	58 (26.4)	83 (37.7)	**220**
Idaho	—	—	15 (9.0)	28 (16.8)	46 (27.5)	71 (42.5)	**167**
Indiana	25 (3.0)	43 (5.1)	62 (7.3)	114 (13.5)	260 (30.7)	342 (40.4)	**846**
Iowa	13 (2.8)	31 (6.8)	56 (12.2)	74 (16.2)	116 (25.3)	168 (36.7)	**458**
Kansas	13 (1.9)	41 (6.1)	50 (7.5)	87 (13.0)	202 (30.1)	277 (41.3)	**670**
Kentucky	16 (4.5)	24 (6.8)	36 (10.2)	43 (12.2)	97 (27.5)	137 (38.8)	**353**
Louisiana	37 (4.3)	71 (8.2)	96 (11.1)	127 (14.7)	233 (26.9)	301 (34.8)	**865**
Maine	5 (2.4)	6 (2.8)	23 (10.9)	35 (16.6)	68 (32.2)	74 (35.1)	**211**
Michigan	84 (3.2)	139 (5.2)	210 (7.9)	381 (14.3)	775 (29.1)	1,074 (40.3)	**2,663**
Minnesota	18 (2.1)	46 (5.3)	63 (7.2)	119 (13.6)	245 (28.1)	381 (43.7)	**872**
Mississippi	13 (4.8)	20 (7.4)	27 (10.0)	38 (14.0)	83 (30.6)	90 (33.2)	**271**
Missouri	14 (2.8)	26 (5.3)	47 (9.5)	53 (10.7)	153 (31.0)	201 (40.7)	**494**
Montana	—	—	25 (12.3)	40 (19.6)	59 (28.9)	70 (34.3)	**204**
Nebraska	7 (3.5)	9 (4.5)	16 (8.0)	35 (17.4)	66 (32.8)	68 (33.8)	**201**
Nevada	12 (1.9)	30 (4.6)	65 (10.0)	117 (18.1)	160 (24.7)	264 (40.7)	**648**
New Jersey^††^	53 (2.3)	140 (6.1)	248 (10.8)	380 (16.6)	625 (27.3)	841 (36.8)	**2,287**
New Mexico	24 (3.8)	34 (5.3)	85 (13.3)	94 (14.7)	161 (25.2)	240 (37.6)	**638**
New York	241 (2.5)	479 (5.0)	875 (9.2)	1,646 (17.2)	2,642 (27.7)	3,661 (38.4)	**9,544**
New York City	153 (2.6)	292 (4.9)	573 (9.6)	1,029 (17.3)	1,598 (26.9)	2,304 (38.7)	**5,949**
New York State	88 (2.4)	187 (5.2)	302 (8.4)	617 (17.2)	1,044 (29.0)	1,357 (37.7)	**3,595**
North Carolina	70 (2.7)	122 (4.8)	235 (9.2)	385 (15.1)	760 (29.7)	984 (38.5)	**2,556**
North Dakota	6 (5.3)	5 (4.4)	10 (8.8)	11 (9.7)	41 (36.3)	40 (35.4)	**113**
Ohio	73 (3.3)	109 (5.0)	190 (8.7)	316 (14.4)	622 (28.4)	877 (40.1)	**2,187**
Oklahoma	10 (1.9)	29 (5.5)	40 (7.6)	93 (17.7)	152 (29.0)	201 (38.3)	**525**
Oregon	21 (2.3)	35 (3.8)	72 (7.8)	140 (15.2)	257 (27.9)	397 (43.1)	**922**
Pennsylvania	86 (2.8)	168 (5.4)	271 (8.7)	462 (14.8)	910 (29.2)	1,217 (39.1)	**3,114**
Rhode Island	8 (3.2)	12 (4.8)	16 (6.4)	25 (10.0)	71 (28.5)	117 (47.0)	**249**
South Carolina	12 (2.0)	26 (4.2)	47 (7.7)	135 (22.0)	167 (27.2)	227 (37.0)	**614**
South Dakota	—	5 (11.9)	8 (19.0)	—	9 (21.4)	16 (38.1)	**42**
Tennessee	34 (3.1)	72 (6.5)	119 (10.7)	140 (12.6)	306 (27.6)	438 (39.5)	**1,109**
Texas	151 (2.9)	253 (4.9)	446 (8.7)	703 (13.6)	1,522 (29.5)	2,077 (40.3)	**5,152**
Utah	0 (0.0)	8 (2.2)	28 (7.7)	45 (12.4)	112 (30.9)	170 (46.8)	**363**
Vermont	—	7 (5.4)	15 (11.6)	—	33 (25.6)	52 (40.3)	**129**
Virginia	38 (2.4)	68 (4.3)	133 (8.3)	196 (12.3)	454 (28.4)	707 (44.3)	**1,596**
Washington	36 (1.9)	88 (4.5)	199 (10.3)	322 (16.6)	562 (29.0)	734 (37.8)	**1,941**
West Virginia	6 (4.0)	11 (7.3)	12 (7.9)	19 (12.6)	40 (26.5)	63 (41.7)	**151**
Wisconsin**	13 (2.0)	41 (6.4)	68 (10.7)	92 (14.5)	167 (26.3)	255 (40.1)	**636**
**Total**	**1,362 (2.7)**	**2,649 (5.2)**	**4,659 (9.2)**	**7,715 (15.2)**	**14,380 (28.3)**	**20,054 (39.5)**	**50,819**
**Abortion rate** ^§§^	**0.5**	**1.7**	**3.1**	**5.2**	**9.6**	**13.2**	—
**Abortion ratio** ^¶¶^	**684**	**447**	**327**	**285**	**292**	**247**	—

* Data from 45 reporting areas; excludes seven reporting areas (California, Florida, Illinois, Maryland, Massachusetts, New Hampshire, and Wyoming) that did not report, did not report age among adolescents by individual year, or did not meet reporting standards.

^†^ Percentages for the individual component categories might not add to 100 because of rounding.

^§^ Cells with a value in the range of 1–4 or cells that would allow for calculation of these small values have been suppressed.

^¶^ Because reporting is not mandatory, information could not be obtained for all abortions performed in the District of Columbia.

** Includes residents only.

^††^ Data from hospitals and licensed ambulatory care facilities only; because reporting is not mandatory for private physicians and women's centers, information could not be obtained for all abortions performed in New Jersey.

^§§^ Number of abortions obtained by adolescents in a given age group per 1,000 adolescents in that same age group. Adolescents aged 13–14 years were used as the denominator for adolescents aged <15 years. For each reporting area, abortions for women of unknown age were distributed according to the distribution of abortions among women of known age for that area.

^¶¶^ Number of abortions obtained by adolescents in a given age group per 1,000 live births to adolescents in that same age group. For each reporting area, abortions for women of unknown age were distributed according to the distribution of abortions among women of known age for that area.

**TABLE 6:** Reported abortions among adolescents, by known age and year — selected reporting areas,* United States, 2006–2015

Age (yrs)	Year										% change			
	2006	2007	2008	2009	2010	2011	2012	2013	2014	2015	2006 to 2010	2011 to 2015	2014 to 2015	2006 to 2015
**% of abortions**														
<15	3.1	3.1	3.1	3.0	3.1	3.0	3.1	2.9	2.8	2.7	0.0	-10.0	-3.6	**-12.9**
15	6.1	6.0	5.8	5.6	5.8	5.6	5.6	5.2	5.2	5.2	-4.9	-7.1	0.0	**-14.8**
16	11.7	11.4	10.8	10.6	10.4	10.1	9.9	9.5	9.5	9.1	-11.1	-9.9	-4.2	**-22.2**
17	17.3	17.4	17.2	16.9	16.4	16.1	15.7	15.2	15.1	15.2	-5.2	-5.6	0.7	**-12.1**
18	27.9	28.1	28.3	28.0	27.6	28.1	27.7	28.1	28.1	28.3	-1.1	0.7	0.7	**1.4**
19	33.9	34.0	34.8	35.8	36.7	37.2	38.1	39.2	39.2	39.5	8.3	6.2	0.8	**16.5**
**Abortion rate^†^**														
<15	1.2	1.2	1.1	1.0	1.0	0.8	0.7	0.6	0.5	0.5	-16.7	-37.5	0.0	**-58.3**
15	4.5	4.4	4.2	3.8	3.6	3.1	2.6	2.2	1.9	1.7	-20.0	-45.2	-10.5	**-62.2**
16	8.5	8.2	7.7	7.0	6.3	5.5	4.7	4.0	3.6	3.1	-25.9	-43.6	-13.9	**-63.5**
17	13.1	12.4	12.2	11.1	9.9	8.6	7.3	6.3	5.6	5.2	-24.4	-39.5	-7.1	**-60.3**
18	21.2	20.6	19.5	17.9	16.1	14.8	12.6	11.6	10.5	9.7	-24.1	-34.5	-7.6	**-54.2**
19	25.7	25.0	24.6	22.3	21.0	18.9	17.0	15.7	14.2	13.3	-18.3	-29.6	-6.3	**-48.2**
**Abortion ratio^§^**														
<15	740	758	786	801	826	813	769	770	727	693	11.6	-14.8	-4.7	**-6.4**
15	517	493	510	494	529	507	469	451	460	448	2.3	-11.6	-2.6	**-13.3**
16	422	406	390	384	386	375	346	340	344	328	-8.5	-12.5	-4.7	**-22.3**
17	345	337	332	324	324	320	294	288	283	288	-6.1	-10.0	1.8	**-16.5**
18	349	336	336	321	326	325	299	300	293	295	-6.6	-9.2	0.7	**-15.5**
19	302	290	294	287	292	281	262	260	253	250	-3.3	-11.0	-1.2	**-17.2**
**Total (no.)**	**111,491**	**109,693**	**107,671**	**98,784**	**89,979**	**78,845**	**67,707**	**60,221**	**53,967**	**49,526**	**—**	**—**	**—**	**—**

* Data from 40 reporting areas; by year, these areas represent 87%–93% of all abortions reported to CDC for adolescents during 2006–2015. Excludes 12 reporting areas (California, District of Columbia, Florida, Illinois, Louisiana, Maine, Maryland, Massachusetts, New Hampshire, Rhode Island, Vermont, and Wyoming) that did not report, did not report age among adolescents by individual year, or did not meet reporting standards for ≥1 year.

^†^ Number of abortions obtained by adolescents in a given age group per 1,000 adolescents in that same age group. Adolescents aged 13–14 years were used as the denominator for adolescents aged <15 years. For each reporting area, abortions for women of unknown age were distributed according to the distribution of abortions among women of known age for that area.

^§^ Number of abortions obtained by adolescents in a given age group per 1,000 live births to adolescents in that same age group. For each reporting area, abortions for women of unknown age were distributed according to the distribution of abortions among women of known age for that area.

In 2015, the abortion ratio for adolescents was highest among adolescents aged <15 years (684 abortions per 1,000 live births among adolescents aged <15 years) and was lowest among adolescents aged ≥17 years (285, 292, and 247 abortions per 1,000 live births among adolescents aged 17, 18, and 19 years, respectively) (Table 5). During 2006–2015 and 2011–2015, abortion ratios decreased among adolescents of all ages (Table 6).

### Gestational Age

Among the 40 areas that reported gestational age^§§§^ at the time of abortion for 2015, approximately two thirds (65.4%) of abortions were performed by ≤8 weeks’ gestation, and nearly all (91.1%) were performed at ≤13 weeks’ gestation (Table 7). Few abortions were performed at 14–20 weeks’ gestation (7.6%) or at ≥21 weeks’ gestation (1.3%). Among the 33 reporting areas that provided data on gestational age every year during 2006–2015, the percentage of abortions performed at ≤13 weeks’ gestation declined minimally from 91.5% to 91.0% (Table 8). However, within this gestational age range, a shift occurred toward earlier gestational ages, with the percentage of abortions performed at ≤8 weeks’ gestation increasing 3% and the percentage of abortions performed at 9–13 weeks’ gestation decreasing 9%. For the entire period of analysis, abortions performed at >13 weeks’ gestation accounted for ≤9.0% of abortions.

**TABLE 7:** Reported abortions, by known weeks of gestation* and reporting area of occurrence — selected reporting areas,^†^ United States, 2015

State/Area	Weeks of gestation						Total abortions reported by known gestational age
	≤8	9–13	14–15	16–17	18–20	≥21	
	No. (%)^§^	No. (%)	No. (%)	No. (%)	No. (%)	No. (%)	No. (% of all reported abortions)^¶^
Alabama	3,191 (54.1)	2,074 (35.2)	310 (5.3)	149 (2.5)	130 (2.2)	45 (0.8)	**5,899 (100.0)**
Alaska	888 (66.7)	439 (33.0)	—**	0 (0.0)	—	—	**1,332 (99.9)**
Arizona	8,335 (65.9)	3,198 (25.3)	468 (3.7)	243 (1.9)	249 (2.0)	156 (1.2)	**12,649 (100.0)**
Arkansas^††^	2,065 (54.8)	1,068 (28.3)	232 (6.2)	175 (4.6)	196 (5.2)	35 (0.9)	**3,771 (100.0)**
Colorado	7,300 (72.4)	1,959 (19.4)	299 (3.0)	155 (1.5)	69 (0.7)	298 (3.0)	**10,080 (99.7)**
Delaware	1,628 (58.5)	986 (35.4)	127 (4.6)	26 (0.9)	9 (0.3)	7 (0.3)	**2,783 (99.9)**
Georgia	19,312 (62.3)	8,208 (26.5)	1,069 (3.4)	831 (2.7)	859 (2.8)	730 (2.4)	**31,009 (100.0)**
Hawaii	1,025 (51.3)	752 (37.6)	65 (3.3)	50 (2.5)	75 (3.8)	31 (1.6)	**1,998 (98.6)**
Idaho	832 (65.4)	398 (31.3)	34 (2.7)	8 (0.6)	0 (0.0)	0 (0.0)	**1,272 (100.0)**
Indiana	4,799 (60.3)	3,136 (39.4)	—	—	5 (0.1)	6 (0.1)	**7,955 (100.0)**
Iowa	2,762 (69.3)	968 (24.3)	97 (2.4)	88 (2.2)	62 (1.6)	9 (0.2)	**3,986 (99.9)**
Kansas	4,345 (62.7)	1,970 (28.4)	233 (3.4)	162 (2.3)	176 (2.5)	45 (0.6)	**6,931 (100.0)**
Kentucky	2,004 (62.9)	804 (25.2)	122 (3.8)	105 (3.3)	107 (3.4)	46 (1.4)	**3,188 (100.0)**
Louisiana	6,188 (66.6)	2,428 (26.1)	399 (4.3)	189 (2.0)	76 (0.8)	17 (0.2)	**9,297 (99.3)**
Maine	1,093 (59.5)	648 (35.3)	37 (2.0)	28 (1.5)	—	—	**1,836 (100.0)**
Michigan	16,822 (62.0)	7,210 (26.6)	1,305 (4.8)	745 (2.7)	603 (2.2)	430 (1.6)	**27,115 (99.9)**
Minnesota	6,542 (66.3)	2,381 (24.1)	358 (3.6)	233 (2.4)	225 (2.3)	122 (1.2)	**9,861 (100.0)**
Mississippi	1,740 (66.6)	700 (26.8)	144 (5.5)	29 (1.1)	0 (0.0)	0 (0.0)	**2,613 (100.0)**
Missouri	2,624 (55.1)	1,522 (31.9)	196 (4.1)	154 (3.2)	192 (4.0)	77 (1.6)	**4,765(100.0)**
Montana	1,044 (64.9)	407 (25.3)	62 (3.9)	47 (2.9)	40 (2.5)	8 (0.5)	**1,608 (99.8)**
Nebraska	1,304 (65.1)	544 (27.1)	91 (4.5)	46 (2.3)	19 (0.9)	0 (0.0)	**2,004 (100.0)**
Nevada	4,863 (69.1)	1,696 (24.1)	234 (3.3)	123 (1.7)	90 (1.3)	31 (0.4)	**7,037 (98.9)**
New Jersey^§§^	14,603 (63.8)	4,790 (20.9)	1,191 (5.2)	816 (3.6)	854 (3.7)	640 (2.8)	**22,894 (99.6)**
New Mexico	3,087 (68.3)	761 (16.8)	126 (2.8)	87 (1.9)	121 (2.7)	336 (7.4)	**4,518 (96.8)**
New York City	43,674 (68.7)	13,777 (21.7)	1,908 (3.0)	1,200 (1.9)	1,566 (2.5)	1,485 (2.3)	**63,610 (99.9)**
North Carolina	17,969 (66.7)	6,725 (25.0)	1,097 (4.1)	718 (2.7)	396 (1.5)	17 (0.1)	**26,922 (97.4)**
North Dakota	772 (66.2)	339 (29.1)	45 (3.9)	10 (0.9)	0 (0.0)	0 (0.0)	**1,166 (100.0)**
Ohio	10,910 (52.0)	7,485 (35.7)	1,120 (5.3)	714 (3.4)	602 (2.9)	145 (0.7)	**20,976 (100.0)**
Oklahoma	3,661 (77.7)	976 (20.7)	68 (1.4)	0 (0.0)	—	—	**4,709 (100.0)**
Oregon	5,956 (69.6)	1,904 (22.3)	208 (2.4)	156 (1.8)	182 (2.1)	148 (1.7)	**8,554 (99.3)**
Rhode Island	1,761 (67.0)	656 (25.0)	113 (4.3)	55 (2.1)	33 (1.3)	10 (0.4)	**2,628 (99.2)**
South Carolina	4,143 (71.7)	1,586 (27.4)	21 (0.4)	—	16 (0.3)	—	**5,778 (100.0)**
South Dakota	248 (56.0)	186 (42.0)	—	0 (0.0)	5 (1.1)	—	**443 (99.8)**
Tennessee	7,244 (64.3)	3,573 (31.7)	404 (3.6)	32 (0.3)	7 (0.1)	13 (0.1)	**11,273 (98.8)**
Texas^††^	34,831 (64.6)	14,673 (27.2)	2,207 (4.1)	1,123 (2.1)	855 (1.6)	248 (0.5)	**53,937 (100.0)**
Utah	2,239 (71.3)	671 (21.4)	105 (3.3)	57 (1.8)	39 (1.2)	30 (1.0)	**3,141 (98.9)**
Vermont	906 (71.7)	305 (24.1)	26 (2.1)	7 (0.6)	11 (0.9)	9 (0.7)	**1,264 (99.9)**
Virginia	14,343 (76.9)	3,848 (20.6)	74 (0.4)	123 (0.7)	180 (1.0)	76 (0.4)	**18,644 (99.9)**
Washington	12,028 (70.4)	3,610 (21.1)	488 (2.9)	310 (1.8)	321 (1.9)	323 (1.9)	**17,080 (99.9)**
West Virginia	918 (60.6)	499 (32.9)	56 (3.7)	28 (1.8)	—	—	**1,516 (100.0)**
**Total**	**279,999 (65.4)**	**109,860 (25.7)**	**15,146 (3.5)**	**9,030 (2.1)**	**8,410 (2.0)**	**5,597 (1.3)**	**428,042** **(99.6)** ^¶¶^

* Gestational age based on the clinician's estimate (Alabama, Alaska, Arizona, Colorado, Delaware, Georgia, Hawaii, Idaho, Indiana, Iowa, Kansas, Kentucky, Louisiana, Maine, Michigan, Minnesota, Mississippi, Missouri, Montana, Nebraska, Nevada, New Jersey, New Mexico, New York City, North Carolina, North Dakota, Ohio, Oregon, Rhode Island, South Carolina, South Dakota, Tennessee, Utah, Vermont, Washington, and West Virginia); gestational age calculated from the last normal menstrual period (Oklahoma); clinician’s estimate of gestation based on estimated date of conception (Virginia); probable postfertilization age (Arkansas and Texas).

^†^ Data are from 40 reporting areas; excludes 12 areas (California, Connecticut, District of Columbia, Florida, Illinois, Maryland, Massachusetts, New Hampshire, New York State, Pennsylvania, Wisconsin, and Wyoming) that did not report, did not report by gestational age, or did not meet reporting standards.

^§^ Percentages for the individual component categories might not add to 100 because of rounding.

^¶^ Percentage is calculated as the number of abortions reported by known gestational age divided by the sum of abortions reported by known and unknown gestational age.

** Cells with a value in the range of 1–4 or cells that would allow for calculation of these small values have been suppressed.

^††^ Two weeks were added to the probable postfertilization age to provide a corresponding measure to gestational age based on the clinician’s estimate.

^§§^ Data from hospitals and licensed ambulatory care facilities only; because reporting is not mandatory for private physicians and women's centers, information could not be obtained for all abortions performed in New Jersey.

^¶¶^ Percentage based on a total of 429,587 abortions reported among the areas that met reporting standards for gestational age.

**TABLE 8:** Reported abortions, by known weeks of gestation and year — selected reporting areas,* United States, 2006–2015

Weeks of gestation	Year										% change			
	2006	2007	2008	2009	2010	2011	2012	2013	2014	2015	2006 to 2010	2011 to 2015	2014 to 2015	2006 to 2015
**≤13 weeks’ gestation (%)**	**91.5**	**91.5**	**91.5**	**91.9**	**91.9**	**91.5**	**91.4**	**91.6**	**91.0**	**91.0**	**0.4**	**-0.5**	**0.0**	**-0.5**
≤8	63.5	63.7	64.2	65.3	65.9	65.7	65.8	65.9	64.8	65.4	3.8	-0.5	0.9	**3.0**
9–13	28.0	27.8	27.3	26.6	26.0	25.8	25.6	25.7	26.2	25.6	-7.1	-0.8	-2.3	**-8.6**
**>13 weeks' gestation (%)**	**8.4**	**8.5**	**8.5**	**8.2**	**8.2**	**8.5**	**8.6**	**8.5**	**9.0**	**9.0**	**-2.4**	**5.9**	**0.0**	**7.1**
14–15	3.3	3.4	3.4	3.3	3.3	3.4	3.5	3.4	3.5	3.5	0.0	2.9	0.0	**6.1**
16–17	1.8	1.9	1.9	1.8	1.8	1.8	1.9	1.9	2.2	2.1	0.0	16.7	-4.5	**16.7**
18–20	1.9	1.9	1.9	1.8	1.8	1.9	1.9	1.9	1.9	2.0	-5.3	5.3	5.3	**5.3**
≥21	1.4	1.3	1.3	1.3	1.3	1.4	1.3	1.3	1.4	1.4	-7.1	0.0	0.0	**0.0**
**Total (no.)**	**536,848**	**530,632**	**533,302**	**510,891**	**501,176**	**474,584**	**449,983**	**429,825**	**418,587**	**407,877**	**—**	**—**	**—**	**—**

* Data from 33 reporting areas; by year, these reporting areas represent 76%–85% of the abortions reported to CDC by gestational age during 2006–2015. Excludes 19 areas (California, Connecticut, Delaware, District of Columbia, Florida, Illinois, Maine, Maryland, Massachusetts, Mississippi, Nebraska, Nevada, New Hampshire, New York State, Pennsylvania, Rhode Island, Vermont, Wisconsin, and Wyoming) that did not report, did not report by gestational age, or did not meet reporting standards for ≥1 year.

Among abortions performed at ≤13 weeks’ gestation and reported by individual week of gestation for 2015, 37.6% were performed at ≤6 weeks’ gestation (Table 9). The percentage contribution to abortions performed at ≤13 weeks’ gestation was progressively smaller for each additional week of gestation: 19.6% were performed at 7 weeks’ gestation, and 3.1% were performed at 13 weeks’ gestation. Among the 33 areas that reported by exact week of gestation for abortions performed at ≤13 weeks’ gestation every year during 2006–2015, a shift occurred toward the earliest gestational age reported: abortions performed at ≤6 weeks’ gestation increased 11%, those performed at 7–12 weeks’ gestation decreased 3%–15%, and those performed at 13 weeks’ gestation were stable (Table 10).

**TABLE 9:** Reported abortions obtained at ≤13 weeks’ gestation,* by weeks of gestation and reporting area of occurrence — selected reporting areas,^†^ United States, 2015

State/Area	Weeks of gestation								Total no. of abortions at ≤13 weeks
	≤6	7	8	9	10	11	12	13	
	No. (%)^§^	No. (%)	No. (%)	No. (%)	No. (%)	No. (%)	No. (%)	No. (%)	
Alabama	1,131 (21.5)	1,091 (20.7)	969 (18.4)	640 (12.2)	507 (9.6)	372 (7.1)	275 (5.2)	280 (5.3)	**5,265**
Alaska	387 (29.2)	259 (19.5)	242 (18.2)	153 (11.5)	87 (6.6)	54 (4.1)	72 (5.4)	73 (5.5)	**1,327**
Arizona	3,699 (32.1)	2,676 (23.2)	1,960 (17.0)	1,117 (9.7)	743 (6.4)	681 (5.9)	343 (3.0)	314 (2.7)	**11,533**
Arkansas^¶^	938 (29.9)	591 (18.9)	536 (17.1)	384 (12.3)	245 (7.8)	214 (6.8)	109 (3.5)	116 (3.7)	**3,133**
Colorado	4,113 (44.4)	1,882 (20.3)	1,305 (14.1)	731 (7.9)	464 (5.0)	340 (3.7)	194 (2.1)	230 (2.5)	**9,259**
Delaware	668 (25.6)	518 (19.8)	442 (16.9)	393 (15.0)	210 (8.0)	164 (6.3)	122 (4.7)	97 (3.7)	**2,614**
Georgia	9,272 (33.7)	6,008 (21.8)	4,032 (14.7)	2,659 (9.7)	1,826 (6.6)	1,558 (5.7)	1,304 (4.7)	861 (3.1)	**27,520**
Hawaii	410 (23.1)	302 (17.0)	313 (17.6)	248 (14.0)	155 (8.7)	125 (7.0)	112 (6.3)	112 (6.3)	**1,777**
Idaho	330 (26.8)	270 (22.0)	232 (18.9)	152 (12.4)	96 (7.8)	65 (5.3)	39 (3.2)	46 (3.7)	**1,230**
Indiana	1,454 (18.3)	1,733 (21.8)	1,612 (20.3)	986 (12.4)	687 (8.7)	575 (7.2)	444 (5.6)	444 (5.6)	**7,935**
Iowa	1,348 (36.1)	790 (21.2)	624 (16.7)	306 (8.2)	209 (5.6)	176 (4.7)	157 (4.2)	120 (3.2)	**3,730**
Kansas	2,120 (33.6)	1,320 (20.9)	905 (14.3)	699 (11.1)	423 (6.7)	367 (5.8)	282 (4.5)	199 (3.2)	**6,315**
Kentucky	847 (30.2)	661 (23.5)	496 (17.7)	300 (10.7)	169 (6.0)	155 (5.5)	101 (3.6)	79 (2.8)	**2,808**
Louisiana	3,098 (36.0)	1,909 (22.2)	1,181 (13.7)	835 (9.7)	501 (5.8)	464 (5.4)	333 (3.9)	295 (3.4)	**8,616**
Maine	327 (18.8)	422 (24.2)	344 (19.8)	225 (12.9)	154 (8.8)	90 (5.2)	109 (6.3)	70 (4.0)	**1,741**
Michigan	8,092 (33.7)	4,791 (19.9)	3,939 (16.4)	2,692 (11.2)	1,390 (5.8)	1,130 (4.7)	1,100 (4.6)	898 (3.7)	**24,032**
Minnesota	3,378 (37.9)	1,792 (20.1)	1,372 (15.4)	935 (10.5)	511 (5.7)	428 (4.8)	288 (3.2)	219 (2.5)	**8,923**
Mississippi	804 (33.0)	573 (23.5)	363 (14.9)	256 (10.5)	137 (5.6)	138 (5.7)	90 (3.7)	79 (3.2)	**2,440**
Missouri	904 (21.8)	991 (23.9)	729 (17.6)	588 (14.2)	303 (7.3)	326 (7.9)	192 (4.6)	113 (2.7)	**4,146**
Montana	591 (40.7)	240 (16.5)	213 (14.7)	153 (10.5)	78 (5.4)	67 (4.6)	62 (4.3)	47 (3.2)	**1,451**
Nebraska	920 (49.8)	179 (9.7)	205 (11.1)	150 (8.1)	110 (6.0)	108 (5.8)	107 (5.8)	69 (3.7)	**1,848**
Nevada	2,589 (39.5)	1,291 (19.7)	983 (15.0)	740 (11.3)	371 (5.7)	308 (4.7)	143 (2.2)	134 (2.0)	**6,559**
New Jersey**	8,421 (43.4)	3,717 (19.2)	2,465 (12.7)	1,596 (8.2)	1,008 (5.2)	654 (3.4)	796 (4.1)	736 (3.8)	**19,393**
New Mexico	2,099 (54.5)	571 (14.8)	417 (10.8)	238 (6.2)	195 (5.1)	128 (3.3)	120 (3.1)	80 (2.1)	**3,848**
New York City	24,560 (42.7)	11,101 (19.3)	8,013 (13.9)	5,120 (8.9)	3,234 (5.6)	2,407 (4.2)	1,889 (3.3)	1,127 (2.0)	**57,451**
North Carolina	8,741 (35.4)	5,161 (20.9)	4,067 (16.5)	2,315 (9.4)	1,489 (6.0)	1,139 (4.6)	962 (3.9)	820 (3.3)	**24,694**
North Dakota	382 (34.4)	227 (20.4)	163 (14.7)	139 (12.5)	67 (6.0)	55 (5.0)	44 (4.0)	34 (3.1)	**1,111**
Ohio	4,500 (24.5)	3,534 (19.2)	2,876 (15.6)	2,367 (12.9)	1,743 (9.5)	1,468 (8.0)	1,054 (5.7)	853 (4.6)	**18,395**
Oklahoma	2,857 (61.6)	422 (9.1)	382 (8.2)	339 (7.3)	176 (3.8)	222 (4.8)	174 (3.8)	65 (1.4)	**4,637**
Oregon	3,376 (43.0)	1,452 (18.5)	1,128 (14.4)	669 (8.5)	394 (5.0)	304 (3.9)	284 (3.6)	253 (3.2)	**7,860**
Rhode Island	1,088 (45.0)	414 (17.1)	259 (10.7)	231 (9.6)	138 (5.7)	131 (5.4)	61 (2.5)	95 (3.9)	**2,417**
South Carolina	2,192 (38.3)	1,068 (18.6)	883 (15.4)	516 (9.0)	372 (6.5)	347 (6.1)	207 (3.6)	144 (2.5)	**5,729**
South Dakota	88 (20.3)	88 (20.3)	72 (16.6)	71 (16.4)	34 (7.8)	27 (6.2)	16 (3.7)	38 (8.8)	**434**
Tennessee	3,353 (31.0)	2,183 (20.2)	1,708 (15.8)	1,217 (11.3)	789 (7.3)	771 (7.1)	415 (3.8)	381 (3.5)	**10,817**
Texas^¶^	19,450 (39.3)	8,851 (17.9)	6,530 (13.2)	5,024 (10.1)	3,562 (7.2)	2,835 (5.7)	1,707 (3.4)	1,545 (3.1)	**49,504**
Utah	1,255 (43.1)	582 (20.0)	402 (13.8)	230 (7.9)	156 (5.4)	139 (4.8)	89 (3.1)	57 (2.0)	**2,910**
Vermont	402 (33.2)	281 (23.2)	223 (18.4)	126 (10.4)	63 (5.2)	53 (4.4)	33 (2.7)	30 (2.5)	**1,211**
Virginia	9,479 (52.1)	2,909 (16.0)	1,955 (10.7)	1,393 (7.7)	930 (5.1)	766 (4.2)	479 (2.6)	280 (1.5)	**18,191**
Washington	6,512 (41.6)	3,180 (20.3)	2,336 (14.9)	1,203 (7.7)	789 (5.0)	700 (4.5)	441 (2.8)	477 (3.1)	**15,638**
West Virginia	351 (24.8)	335 (23.6)	232 (16.4)	161 (11.4)	108 (7.6)	91 (6.4)	103 (7.3)	36 (2.5)	**1,417**
**Total**	**146,526(37.6)**	**76,365 (19.6)**	**57,108 (14.6)**	**38,297 (9.8)**	**24,623 (6.3)**	**20,142 (5.2)**	**14,852 (3.8)**	**11,946 (3.1)**	**389,859**

* Gestational age based on the clinician's estimate (Alabama, Alaska, Arizona, Colorado, Delaware, Georgia, Hawaii, Idaho, Indiana, Iowa, Kansas, Kentucky, Louisiana, Maine, Michigan, Minnesota, Mississippi, Missouri, Montana, Nebraska, Nevada, New Jersey, New Mexico, New York City, North Carolina, North Dakota, Ohio, Oregon, Rhode Island, South Carolina, South Dakota, Tennessee, Utah, Vermont, Washington, and West Virginia); gestational age calculated from the last normal menstrual period (Oklahoma); clinician’s estimate of gestation based on estimated date of conception (Virginia); probable postfertilization age (Arkansas and Texas).

^†^ Data are from 40 reporting areas; excludes 12 areas (California, Connecticut, District of Columbia, Florida, Illinois, Maryland, Massachusetts, New Hampshire, New York State, Pennsylvania, Wisconsin, and Wyoming) that did not report, did not report by gestational age, or did not meet reporting standards.

^§^ Percentages for the individual component categories might not add to 100 because of rounding.

^¶^ Two weeks were added to the probable postfertilization age to provide a corresponding measure to gestational age based on the clinician’s estimate.

** Data from hospitals and licensed ambulatory care facilities only; because reporting is not mandatory for private physicians and women's centers, information could not be obtained for all abortions performed in New Jersey.

**TABLE 10:** Reported abortions obtained at ≤13 weeks’ gestation, by weeks of gestation and year — selected reporting areas,* United States, 2006–2015

Weeks of gestation	Year										% change			
	2006	2007	2008	2009	2010	2011	2012	2013	2014	2015	2006 to 2010	2011 to 2015	2014 to 2015	2006 to 2015
**% distribution among abortions reported at ≤13 weeks**														
≤6	34.0	35.0	35.5	36.7	37.9	37.6	38.4	37.9	37.1	37.7	11.5	0.3	1.6	**10.9**
7	20.1	20.0	19.8	19.4	19.3	19.6	19.3	19.5	19.3	19.6	-4.0	0.0	1.6	**-2.5**
8	15.2	14.7	14.8	14.9	14.5	14.6	14.3	14.6	14.9	14.6	-4.6	0.0	-2.0	**-3.9**
9	10.4	10.2	10.0	9.7	9.8	9.5	9.4	9.4	9.8	9.8	-5.8	3.2	0.0	**-5.8**
10	7.4	7.4	7.1	6.8	6.7	6.5	6.4	6.4	6.6	6.3	-9.5	-3.1	-4.5	**-14.9**
11	5.4	5.4	5.5	5.3	5.1	5.2	5.2	5.1	5.4	5.2	-5.6	0.0	-3.7	**-3.7**
12	4.3	4.3	4.2	4.1	3.9	4.0	3.9	4.0	3.9	3.8	-9.3	-5.0	-2.6	**-11.6**
13	3.1	3.1	3.0	2.9	2.9	3.0	3.1	3.1	3.1	3.1	-6.5	3.3	0.0	**0.0**
**Total (no.)**	**491,630**	**485,709**	**487,837**	**469,055**	**460,424**	**434,216**	**411,526**	**393,570**	**380,683**	**371,029**	**—**	**—**	**—**	**—**

* Data from 33 reporting areas; by year, these reporting areas represent 85%–92% of the abortions reported to CDC at ≤13 weeks’ gestation during 2006–2015. Excludes 19 reporting areas (California, Connecticut, Delaware, District of Columbia, Florida, Illinois, Maine, Maryland, Massachusetts, Mississippi, Nebraska, Nevada, New Hampshire, New York State, Pennsylvania, Rhode Island, Vermont, Wisconsin, and Wyoming) that did not report, did not report by gestational age, or did not meet reporting standards for ≥1 year.

### Method Type

Among the 43 areas that reported by method type for 2015 and included medical abortion on their reporting form, 64.3% of abortions were surgical abortions at ≤13 weeks’ gestation, 24.6% were early medical abortions (a nonsurgical abortion at ≤8 weeks’ gestation), and 8.8% were surgical abortions at >13 weeks’ gestation; other methods (medical abortion at >8 weeks’ gestation, intrauterine instillation, and hysterectomy/hysterotomy) were uncommon (≤2.2%) (Table 11). Among the 34 reporting areas^¶¶¶^ that included medical abortion on their reporting form and provided these data for the relevant years of comparison (2006 versus 2015, 2006 versus 2010, 2011 versus 2015, and 2014 versus 2015), use of early medical abortion increased 8% from 2014 to 2015 (from 22.5% of abortions to 24.2%); from 2006 to 2015, use of early medical abortion increased 114% (from 11.3% of abortions to 24.2%). Increases in early medical abortion occurred both from 2006 to 2010 (from 11.3% of abortions to 18.4% [63% increase]) and from 2011 to 2015 (from 19.2% of abortions to 24.2% [26% increase]).

**TABLE 11:** Reported abortions, by known method type and reporting area of occurrence — selected reporting areas,* United States, 2015

State/Area	Surgical^†^			Medical			Intrauterine instillation^§^	Hysterectomy/Hysterotomy	Total abortions reported by known method type
	Surgical, ≤13 weeks’ gestation	Surgical, >13 weeks’ gestation	Surgical, unknown gestational age	Medical, ≤8 weeks’ gestation	Medical, >8 weeks’ gestation	Medical, unknown gestational age			
	No. (%)^¶^	No. (%)	No. (%)	No. (%)	No. (%)	No. (%)	No. (%)	No. (%)	No. (% of all reported abortions)**
Alabama	3,355 (56.9)	614 (10.4)	0 (0.0)	1,831 (31.0)	95 (1.6)	0 (0.0)	—^††^	—	**5,897 (100.0)**
Alaska	984 (73.9)	—	—	302 (22.7)	38 (2.9)	—	—	0 (0.0)	**1,331 (99.8)**
Arizona	7,494 (59.3)	1,089 (8.6)	—	3,868 (30.6)	178 (1.4)	—	—	0 (0.0)	**12,636 (99.8)**
Arkansas	2,552 (67.7)	638 (16.9)	0 (0.0)	548 (14.5)	33 (0.9)	0 (0.0)	0 (0.0)	0 (0.0)	**3,771 (100.0)**
Colorado	4,826 (49.1)	539 (5.5)	23 (0.2)	4,222 (42.9)	212 (2.2)	10 (0.1)	—	—	**9,833 (97.2)**
Connecticut^§§^	NA	NA	5,544 (55.8)	NA	NA	4,392 (44.2)	—	—	**9,938 (100.0)**
Delaware	1,415 (51.0)	164 (5.9)	—	1,031 (37.1)	163 (5.9)	0 (0.0)	—	0 (0.0)	**2,776 (99.7)**
District of Columbia^¶¶^	845 (66.7)	159 (12.5)	0 (0.0)	0 (0.0)	22 (1.7)	241 (19.0)	0 (0.0)	0 (0.0)	**1,267 (100.0)**
Georgia	19,758 (64.1)	3,478 (11.3)	0 (0.0)	7,186 (23.3)	382 (1.2)	0 (0.0)	0 (0.0)	0 (0.0)	**30,804 (99.3)**
Idaho	670 (52.7)	41 (3.2)	0 (0.0)	497 (39.1)	64 (5.0)	0 (0.0)	0 (0.0)	0 (0.0)	**1,272 (100.0)**
Illinois^§§^	NA	NA	24,811 (72.2)	NA	NA	9,514 (27.7)	53 (0.2)	0 (0.0)	**34,378 (86.3)**
Indiana	5,714 (71.8)	20 (0.3)	—	2,115 (26.6)	106 (1.3)	—	0 (0.0)	0 (0.0)	**7,957 (100.0)**
Iowa	1,546 (38.8)	254 (6.4)	—	2,076 (52.2)	101 (2.5)	—	0 (0.0)	0 (0.0)	**3,980 (99.8)**
Kansas	3,223 (46.5)	612 (8.8)	0 (0.0)	2,673 (38.6)	419 (6.0)	0 (0.0)	0 (0.0)	0 (0.0)	**6,927 (99.9)**
Kentucky	1,597 (50.1)	373 (11.7)	0 (0.0)	1,157 (36.3)	61 (1.9)	0 (0.0)	0 (0.0)	0 (0.0)	**3,188 (100.0)**
Maine	1,209 (65.8)	85 (4.6)	0 (0.0)	417 (22.7)	125 (6.8)	0 (0.0)	0 (0.0)	0 (0.0)	**1,836 (100.0)**
Massachusetts^§§^	NA	NA	12,933 (70.3)	NA	NA	5,324 (28.9)	145 (0.8)	0 (0.0)	**18,402 (99.1)**
Michigan	16,523 (61.0)	3,023 (11.2)	29 (0.1)	6,737 (24.9)	775 (2.9)	5 (0.0)	—	—	**27,095 (99.8)**
Minnesota	5,774 (58.6)	920 (9.3)	0 (0.0)	2,833 (28.7)	326 (3.3)	0 (0.0)	—	—	**9,861 (100.0)**
Mississippi	1,232 (47.1)	172 (6.6)	0 (0.0)	1,057 (40.5)	152 (5.8)	0 (0.0)	0 (0.0)	0 (0.0)	**2,613 (100.0)**
Missouri	2,891 (60.7)	609 (12.8)	0 (0.0)	1,065 (22.4)	198 (4.2)	0 (0.0)	—	—	**4,764 (100.0)**
Montana	672 (41.7)	157 (9.7)	—	724 (44.9)	55 (3.4)	0 (0.0)	0 (0.0)	—	**1,611 (100.0)**
Nebraska	885 (44.2)	154 (7.7)	0 (0.0)	922 (46.0)	43 (2.1)	0 (0.0)	0 (0.0)	0 (0.0)	**2,004 (100.0)**
Nevada	4,938 (72.7)	263 (3.9)	41 (0.6)	1,456 (21.4)	64 (0.9)	31 (0.5)	0 (0.0)	0 (0.0)	**6,793 (95.5)**
New Jersey***	15,281 (66.5)	3,442 (15.0)	88 (0.4)	3,854 (16.8)	280 (1.2)	—	—	35 (0.2)	**22,988 (100.0)**
New York	59,533 (64.8)	7,332 (8.0)	4,677 (5.1)	15,667 (17.1)	3,107 (3.4)	1,462 (1.6)	71 (0.1)	11 (0.0)	**91,860 (98.7)**
New York City	46,636 (73.4)	5,883 (9.3)	25 (0.0)	10,267 (16.2)	644 (1.0)	9 (0.0)	45 (0.1)	11 (0.0)	**63,520 (99.8)**
New York State	12,897 (45.5)	1,449 (5.1)	4,652 (16.4)	5,400 (19.1)	2,463 (8.7)	1,453 (5.1)	26 (0.1)	0 (0.0)	**28,340 (96.2)**
North Carolina	13,606 (51.1)	2,118 (7.9)	194 (0.7)	9,721 (36.5)	577 (2.2)	428 (1.6)	0 (0.0)	0 (0.0)	**26,644 (96.4)**
North Dakota	1,065 (91.3)	54 (4.6)	0 (0.0)	30 (2.6)	15 (1.3)	0 (0.0)	—	—	**1,166 (100.0)**
Ohio	17,240 (82.2)	2,556 (12.2)	0 (0.0)	1,154 (5.5)	26 (0.1)	0 (0.0)	0 (0.0)	0 (0.0)	**20,976 (100.0)**
Oklahoma	2,792 (59.6)	66 (1.4)	0 (0.0)	1,822 (38.9)	—	0 (0.0)	—	0 (0.0)	**4,686 (99.5)**
Oregon	4,675 (54.4)	658 (7.7)	40 (0.5)	3,045 (35.4)	162 (1.9)	15 (0.2)	—	—	**8,598 (99.9)**
Pennsylvania	16,651 (52.3)	3,846 (12.1)	0 (0.0)	10,033 (31.5)	1,281 (4.0)	0 (0.0)	0 (0.0)	0 (0.0)	**31,811 (100.0)**
Rhode Island	1,615 (61.4)	206 (7.8)	11 (0.4)	714 (27.2)	73 (2.8)	10 (0.4)	0 (0.0)	0 (0.0)	**2,629 (99.2)**
South Carolina	3,022 (52.3)	37 (0.6)	0 (0.0)	2,585 (44.8)	127 (2.2)	0 (0.0)	—	—	**5,775 (99.9)**
South Dakota	266 (59.9)	—	—	138 (31.1)	38 (8.6)	0 (0.0)	0 (0.0)	0 (0.0)	**444 (100.0)**
Texas	43,545 (80.7)	4,369 (8.1)	—	5,917 (11.0)	86 (0.2)	—	—	—	**53,926 (100.0)**
Utah	1,822 (57.6)	216 (6.8)	11 (0.3)	1,005 (31.8)	83 (2.6)	24 (0.8)	0 (0.0)	0 (0.0)	**3,161 (99.5)**
Vermont	587 (46.6)	48 (3.8)	—	548 (43.5)	77 (6.1)	0 (0.0)	0 (0.0)	—	**1,261 (99.7)**
Virginia	13,050 (70.0)	437 (2.3)	12 (0.1)	5,060 (27.2)	69 (0.4)	7 (0.0)	—	—	**18,636 (99.9)**
Washington	10,005 (58.5)	1,435 (8.4)	11 (0.1)	5,352 (31.3)	281 (1.6)	7 (0.0)	0 (0.0)	0 (0.0)	**17,091 (100.0)**
West Virginia	1,212 (79.9)	93 (6.1)	0 (0.0)	199 (13.1)	12 (0.8)	0 (0.0)	0 (0.0)	0 (0.0)	**1,516 (100.0)**
Wisconsin^§§,†††^	NA	NA	4,323 (79.2)	NA	NA	1,138 (20.8)	NA	NA	**5,461 (96.5)**
**Total**	**340,475 (64.3)**	**46,637 (8.8)**	—**^§§§^**	**130,309 (24.6)**	**11,785 (2.2)**	—**^¶¶¶^**	**298 (0.1)**	**59 (0.0)**	**529,563** (98.3)****

**Abbreviation:** NA = not available.

* Data from 43 reporting areas; excludes nine reporting areas (California, Florida, Hawaii, Louisiana, Maryland, New Hampshire, New Mexico, Tennessee, and Wyoming) that did not report, did not report by method type, or did not meet reporting standards.

^†^ Includes aspiration curettage, suction curettage, manual vacuum aspiration, menstrual extraction, sharp curettage, and dilation and evacuation procedures.

^§^ Intrauterine instillations reported at ≤12 weeks' gestation are not presented with abortions reported by known method type.

^¶^ Percentages for the individual component categories might not add to 100 because of rounding.

** Percentage is calculated as the number of abortions reported by known method type divided by the sum of abortions reported by known and unknown method type.

^††^ Cells with a value in the range of 1–4 or cells that would allow for calculation of these small values have been suppressed.

^§§^ Numbers for surgical procedures at ≤13 weeks versus >13 weeks and for medical abortion at ≤8 weeks versus >8 weeks are not presented because gestational age data were not provided or were provided in incompatible categories.

^¶¶^ Because reporting is not mandatory, information could not be obtained for all abortions performed in the District of Columbia.

*** Data from hospitals and licensed ambulatory care facilities only; because reporting is not mandatory for private physicians and women's centers, information could not be obtained for all abortions performed in New Jersey.

^†††^ All abortions were reported as surgical or chemically induced. For this report, all surgical abortions were classified as surgical and all chemical abortions as medical.

^§§§^ Surgical abortions reported without a gestational age were distributed among the surgical abortion categories according to the distribution of surgical abortions at known gestational age.

**^¶¶¶^** Medical abortions reported without a gestational age were distributed among the medical abortion categories according to the distribution of medical abortions at known gestational age.

**** Percentage is based on a total of 538,678 abortions reported among the areas that met reporting standards for method type.

Among the 30 reporting areas that provided data by procedure and individual week of gestational age each year from 2011 to 2015,**** when recent clinical guidelines extended mifepristone use to 70 days’ gestation, the percentage of abortions at 9 completed weeks’ gestation that were reported as medical abortions did not change substantially between 2011, 2012, 2013, and 2014 (5.0%, 5.7%, 6.7%, and 7.7%, respectively) and then increased to 13.0% in 2015. Among the 43^††††^ areas that reported by method type for 2015 and included medical abortion on their reporting form, 26.0% were medical abortions performed at ≤9 weeks’ gestation. Of these medical abortions performed at ≤9 weeks’ gestation, 94.6% were performed at ≤8 weeks and 5.4% were performed at 9 weeks.

In contrast to the increase that occurred in use of early medical abortion, use of surgical abortion at ≤13 weeks’ gestation decreased 18% from 2006 to 2015 (from 79.2% of abortions to 64.7%). Surgical abortion at >13 weeks’ gestation consistently accounted for approximately 8.0%–9.0% of all abortions, and all other methods combined consistently accounted for a limited percentage of abortions (1.4%–2.4%) during 2006–2015.

### Race/Ethnicity

Among the 30 areas that reported cross-classified race/ethnicity data for 2015, non-Hispanic white women and non-Hispanic black women accounted for the largest percentages of all abortions (36.9% and 36.0%, respectively), and Hispanic women and non-Hispanic women in the other race category accounted for smaller percentages (18.5% and 8.7%, respectively) (Table 12). Non-Hispanic white women had the lowest abortion rate (6.8 abortions per 1,000 women aged 15–44 years) and ratio (111 abortions per 1,000 live births) and non-Hispanic black women had the highest abortion rate (25.1 abortions per 1,000 women aged 15–44 years) and ratio (390 abortions per 1,000 live births). Data for 2015 also are reported separately by race and by ethnicity (Tables 13 and 14).

**TABLE 12:** Reported abortions, by known race/ethnicity of women who obtained an abortion and reporting area of occurrence — selected reporting areas,* United States, 2015

State/Area	Non-Hispanic			Hispanic	Total abortions reported by known race/ethnicity
	White	Black	Other		No. (% of all reported abortions)^§^
	No. (%)^†^	No. (%)	No. (%)	No. (%)	
Alabama	1,905 (32.4)	3,490 (59.3)	186 (3.2)	302 (5.1)	**5,883 (99.7)**
Alaska	743 (62.2)	88 (7.4)	337 (28.2)	27 (2.3)	**1,195 (89.6)**
Arizona	5,425 (45.2)	1,090 (9.1)	1,040 (8.7)	4,437 (37.0)	**11,992 (94.8)**
Arkansas	1,631 (45.4)	1,734 (48.3)	5 (0.1)	221 (6.2)	**3,591 (95.2)**
Colorado	5,112 (57.9)	672 (7.6)	698 (7.9)	2,349 (26.6)	**8,831 (87.3)**
Delaware	1,158 (41.7)	1,175 (42.3)	165 (5.9)	282 (10.1)	**2,780 (99.8)**
District of Columbia^¶^	189 (15.8)	857 (71.5)	55 (4.6)	98 (8.2)	**1,199 (94.6)**
Georgia	7,183 (24.7)	18,167 (62.4)	1,572 (5.4)	2,179 (7.5)	**29,101 (93.8)**
Hawaii	394 (21.9)	70 (3.9)	1,120 (62.3)	213 (11.9)	**1,797 (88.7)**
Idaho	897 (78.1)	22 (1.9)	45 (3.9)	184 (16.0)	**1,148 (90.3)**
Indiana	4,384 (55.1)	2,364 (29.7)	565 (7.1)	644 (8.1)	**7,957 (100.0)**
Kansas	3,884 (56.3)	1,477 (21.4)	640 (9.3)	899 (13.0)	**6,900 (99.6)**
Kentucky	2,009 (63.0)	863 (27.1)	144 (4.5)	171 (5.4)	**3,187 (100.0)**
Michigan	11,447 (42.6)	13,323 (49.6)	1,112 (4.1)	992 (3.7)	**26,874 (99.0)**
Minnesota	4,928 (53.7)	2,237 (24.4)	1,390 (15.1)	628 (6.8)	**9,183 (93.1)**
Missouri	2,182 (47.1)	2,001 (43.2)	325 (7.0)	125 (2.7)	**4,633 (97.2)**
Montana	1,424 (88.4)	20 (1.2)	167 (10.4)	0 (0.0)	**1,611 (100.0)**
Nevada	2,750 (41.2)	1,005 (15.1)	1,254 (18.8)	1,662 (24.9)	**6,671 (93.7)**
New Jersey**	6,798 (31.3)	6,908 (31.8)	3,806 (17.5)	4,208 (19.4)	**21,720 (94.5)**
New York City^††^	9,769 (16.3)	25,698 (42.9)	6,183 (10.3)	18,195 (30.4)	**59,845 (94.0)**
Ohio	9,709 (49.7)	7,948 (40.7)	1,142 (5.8)	748 (3.8)	**19,547 (93.2)**
Oregon	5,967 (70.8)	503 (6.0)	841 (10.0)	1,113 (13.2)	**8,424 (97.8)**
South Carolina	2,986 (51.8)	2,306 (40.0)	184 (3.2)	294 (5.1)	**5,770 (99.9)**
South Dakota	311 (70.0)	40 (9.0)	63 (14.2)	30 (6.8)	**444 (100.0)**
Tennessee	4,939 (44.6)	5,353 (48.3)	310 (2.8)	472 (4.3)	**11,074 (97.0)**
Texas^§§^	15,430 (28.7)	14,103 (26.2)	3,648 (6.8)	20,586 (38.3)	**53,767 (99.7)**
Utah	2,020 (65.1)	110 (3.5)	274 (8.8)	697 (22.5)	**3,101 (97.6)**
Vermont	1,102 (89.4)	26 (2.1)	76 (6.2)	28 (2.3)	**1,232 (97.4)**
Virginia	6,946 (39.0)	7,978 (44.8)	1,987 (11.1)	916 (5.1)	**17,827 (95.5)**
West Virginia	1,271 (83.8)	201 (13.3)	30 (2.0)	14 (0.9)	**1,516 (100.0)**
**Total**	**124,893 (36.9)**	**121,829 (36.0)**	**29,364 (8.7)**	**62,714 (18.5)**	**338,800** **(95.9)**^¶¶^
Abortion rate***	**6.8**	**25.1**	**13.5**	**11.2**	**10.9**
**Abortion ratio** ^†††^	**111**	**390**	**222**	**147**	**170**

* Data from 30 reporting areas; excludes 22 reporting areas (California, Connecticut, Florida, Illinois, Iowa, Louisiana, Maine, Maryland, Massachusetts, Mississippi, Nebraska, New Hampshire, New Mexico, New York State, North Carolina, North Dakota, Oklahoma, Pennsylvania, Rhode Island, Washington, Wisconsin, and Wyoming) that did not report, did not report by race/ethnicity, or did not meet reporting standards.

^†^ Percentages for the individual component categories might not add to 100 because of rounding.

^§^ Percentage is calculated as the number of abortions reported by known race/ethnicity divided by the sum of abortions reported by known and unknown race/ethnicity.

^¶^ Because reporting is not mandatory, information could not be obtained for all abortions performed in the District of Columbia.

** Data from hospitals and licensed ambulatory care facilities only; because reporting is not mandatory for private physicians and women’s centers, information could not be obtained for all abortions performed in New Jersey.

^††^ Non-Hispanic categories include abortions for women whose ethnicity was reported as unknown; previous evaluation has shown that most reports without ethnicity are for non-Hispanic women.

^§§^ Reporting form contains only one question for race and ethnicity; therefore, abortions reported for women of white, black, and other races (Asian and Native American) are not explicitly identified as non-Hispanic.

^¶¶^ Percentage is based on a total of 353,128 abortions reported among the areas that met reporting standards for race/ethnicity.

*** Number of abortions obtained by women in a given race/ethnicity group per 1,000 women in that same racial/ethnic group. For each reporting area, abortions for women of unknown race/ethnicity were distributed according to the distribution of abortions among women of known race/ethnicity for that area.

^†††^ Number of abortions obtained by women in a given race/ethnicity group per 1,000 live births to women in that same race/ethnicity group. For each reporting area, abortions for women of unknown race/ethnicity were distributed according to the distribution of abortions among women of known race/ethnicity for that area.

**TABLE 13:** Reported abortions, by known race of women who obtained an abortion and reporting area of occurrence — selected reporting areas,* United States, 2015

State/Area	Race			Total abortions reported by known race
	White	Black	Other	
	No. (%)^†^	No. (%)	No. (%)	No. (% all reported abortions)^§^
Alabama	2,146 (36.4)	3,536 (60.0)	212 (3.6)	**5,894 (99.9)**
Alaska	810 (62.2)	102 (7.8)	390 (30.0)	**1,302 (97.6)**
Arkansas	1,691 (49.3)	1,734 (50.6)	5 (0.1)	**3,430 (91.0)**
Colorado	6,272 (67.2)	799 (8.6)	2,267 (24.3)	**9,338 (92.3)**
Delaware	1,411 (50.7)	1,200 (43.1)	174 (6.2)	**2,785 (100.0)**
District of Columbia^¶^	226 (18.8)	913 (75.9)	64 (5.3)	**1,203 (94.9)**
Georgia	7,726 (27.7)	18,450 (66.2)	1,688 (6.1)	**27,864 (89.9)**
Hawaii	526 (27.6)	84 (4.4)	1,294 (68.0)	**1,904 (94.0)**
Idaho	1,041 (89.4)	28 (2.4)	95 (8.2)	**1,164 (91.5)**
Indiana	4,545 (57.1)	2,396 (30.1)	1,016 (12.8)	**7,957 (100.0)**
Iowa	2,865 (71.9)	608 (15.3)	510 (12.8)	**3,983 (99.8)**
Kansas	4,079 (59.7)	1,514 (22.2)	1,234 (18.1)	**6,827 (98.5)**
Louisiana	2,753 (29.6)	5,763 (62.1)	771 (8.3)	**9,287 (99.2)**
Maine	1,582 (86.3)	103 (5.6)	149 (8.1)	**1,834 (99.9)**
Massachusetts	8,573 (50.0)	2,973 (17.3)	5,613 (32.7)	**17,159 (92.4)**
Michigan	12,086 (45.4)	13,380 (50.2)	1,184 (4.4)	**26,650 (98.2)**
Minnesota	5,283 (55.2)	2,413 (25.2)	1,883 (19.7)	**9,579 (97.1)**
Mississippi	527 (20.3)	2,020 (77.6)	55 (2.1)	**2,602 (99.6)**
Missouri	2,263 (48.8)	2,016 (43.5)	360 (7.8)	**4,639 (97.4)**
Montana	1,424 (88.4)	20 (1.2)	167 (10.4)	**1,611 (100.0)**
Nebraska	1,282 (66.6)	352 (18.3)	291 (15.1)	**1,925 (96.1)**
New Jersey**	8,868 (40.0)	9,041 (40.7)	4,282 (19.3)	**22,191 (96.5)**
North Carolina	9,854 (39.6)	13,695 (55.1)	1,314 (5.3)	**24,863 (90.0)**
North Dakota	805 (69.7)	142 (12.3)	208 (18.0)	**1,155 (99.1)**
Ohio	10,338 (51.5)	8,421 (42.0)	1,298 (6.5)	**20,057 (95.6)**
Oklahoma	2,876 (61.1)	914 (19.4)	917 (19.5)	**4,707 (100.0)**
Oregon	6,588 (79.9)	542 (6.6)	1,111 (13.5)	**8,241 (95.7)**
Pennsylvania	15,820 (50.2)	13,332 (42.3)	2,366 (7.5)	**31,518 (99.1)**
Rhode Island	1,918 (76.8)	419 (16.8)	159 (6.4)	**2,496 (94.2)**
South Carolina	3,271 (56.7)	2,315 (40.1)	187 (3.2)	**5,773 (99.9)**
South Dakota	340 (76.6)	40 (9.0)	64 (14.4)	**444 (100.0)**
Tennessee	5,345 (48.3)	5,361 (48.5)	353 (3.2)	**11,059 (96.9)**
Utah	2,321 (82.9)	119 (4.3)	359 (12.8)	**2,799 (88.1)**
Vermont	1,124 (90.4)	30 (2.4)	89 (7.2)	**1,243 (98.3)**
Virginia	7,623 (41.4)	8,248 (44.8)	2,532 (13.8)	**18,403 (98.6)**
West Virginia	1,281 (84.5)	205 (13.5)	30 (2.0)	**1,516 (100.0)**
Wisconsin^††^	3,426 (65.7)	1,436 (27.5)	352 (6.8)	**5,214 (95.5)**
**Total**	**150,909 (48.6)**	**124,664 (40.1)**	**35,043 (11.3)**	**310,616 (95.8)^§§^**
**Abortion Rate^¶¶^**	**6.4**	**23.0**	**16.5**	**9.9**
Abortion Ratio***	**103**	**347**	**276**	**159**

* Data from 37 reporting areas; excludes 15 areas (Arizona, California, Connecticut, Florida, Illinois, Kentucky, Maryland, Nevada, New Hampshire, New Mexico, New York State, New York City, Texas, Washington, and Wyoming) that did not report, did not report by race, or did not meet reporting standards.

^†^ Percentages for the individual component categories might not add to 100 because of rounding.

^§^ Percentage is calculated as the number of abortions reported by known race, divided by the sum of abortions reported by known and unknown race.

^¶^ Because reporting is not mandatory, information could not be obtained for all abortions performed in the District of Columbia.

** Data from hospitals and licensed ambulatory care facilities only; because reporting is not mandatory for private physicians and women's centers, information could not be obtained for all abortions performed in New Jersey.

^††^ Includes residents only.

^§§^ Percentage is based on a total of 324,391 abortions reported among the areas that met reporting standards for race.

^¶¶^ Number of abortions obtained by women in a given racial group per 1,000 women in that same racial group. For each reporting area, abortions for women of unknown race were distributed according to the distribution of abortions among women of known race for that area.

*** Number of abortions obtained by women in a given racial group per 1,000 live births to women in that same racial group. For each reporting area, abortions for women of unknown race were distributed according to the distribution of abortions among women of known race for that area.

**TABLE 14:** Reported abortions, by known ethnicity of women who obtained an abortion and reporting area of occurrence — selected reporting areas,* United States, 2015

State/Area	Ethnicity		Total abortions reported by known ethnicity
	Hispanic	Non-Hispanic	
	No. (%)^†^	No. (%)	No. (% of all reported abortions)^§^
Alabama	302 (5.1)	5,583 (94.9)	**5,885 (99.8)**
Alaska	27 (2.3)	1,168 (97.7)	**1,195 (89.6)**
Arizona	4,437 (35.1)	8,218 (64.9)	**12,655 (100.0)**
Arkansas	221 (5.9)	3,538 (94.1)	**3,759 (99.7)**
Colorado	2,349 (26.2)	6,602 (73.8)	**8,951 (88.5)**
Delaware	282 (10.1)	2,498 (89.9)	**2,780 (99.8)**
District of Columbia^¶^	98 (8.0)	1,128 (92.0)	**1,226 (96.8)**
Georgia	2,179 (7.2)	28,097 (92.8)	**30,276 (97.6)**
Hawaii	213 (11.2)	1,688 (88.8)	**1,901 (93.8)**
Idaho	184 (15.8)	984 (84.2)	**1,168 (91.8)**
Indiana	644 (8.1)	7,313 (91.9)	**7,957 (100.0)**
Kansas	899 (13.0)	6,032 (87.0)	**6,931 (100.0)**
Kentucky	171 (5.4)	3,017 (94.6)	**3,188 (100.0)**
Michigan	992 (3.7)	26,071 (96.3)	**27,063 (99.7)**
Minnesota	628 (6.8)	8,597 (93.2)	**9,225 (93.6)**
Missouri	125 (2.6)	4,617 (97.4)	**4,742 (99.5)**
Montana	0 (0.0)	1,611 (100.0)	**1,611 (100.0)**
Nevada	1,662 (24.2)	5,194 (75.8)	**6,856 (96.3)**
New Jersey**	4,208 (19.3)	17,606 (80.7)	**21,814 (94.9)**
New Mexico	2,192 (55.2)	1,779 (44.8)	**3,971 (85.1)**
New York	22,295 (23.9)	70,801 (76.1)	**93,096 (100.0)**
New York City^††^	18,195 (28.6)	45,451 (71.4)	**63,646 (100.0)**
New York State	4,100 (13.9)	25,350 (86.1)	**29,450 (100.0)**
Ohio	748 (3.8)	19,057 (96.2)	**19,805 (94.4)**
Oregon	1,113 (12.9)	7,497 (87.1)	**8,610 (100.0)**
Pennsylvania	3,050 (9.7)	28,505 (90.3)	**31,555 (99.2)**
South Carolina	294 (5.1)	5,480 (94.9)	**5,774 (99.9)**
South Dakota	30 (6.8)	414 (93.2)	**444 (100.0)**
Tennessee	472 (4.2)	10,734 (95.8)	**11,206 (98.2)**
Texas^††^	20,586 (38.3)	33,181 (61.7)	**53,767 (99.7)**
Utah	697 (22.2)	2,436 (77.8)	**3,133 (98.6)**
Vermont	28 (2.3)	1,216 (97.7)	**1,244 (98.3)**
Virginia	916 (5.1)	17,030 (94.9)	**17,946 (96.2)**
West Virginia	14 (0.9)	1,502 (99.1)	**1,516 (100.0)**
**Total**	**72,056 (17.5)**	**339,194 (82.5)**	**411,250 (98.1)^§§^**
**Abortion rate** ^¶¶^	**11.2**	**11.3**	**11.3**
Abortion ratio***	**148**	**184**	**177**

* Data from 33 reporting areas; excludes 19 areas (California, Connecticut, Florida, Illinois, Iowa, Louisiana, Maine, Maryland, Massachusetts, Mississippi, Nebraska, New Hampshire, North Carolina, North Dakota, Oklahoma, Rhode Island, Washington, Wisconsin, and Wyoming) that did not report, did not report by ethnicity, or did not meet reporting standards.

^†^ Percentages for the individual component categories might not add to 100 because of rounding.

^§^ Percentage is calculated as the number of abortions reported by known ethnicity divided by the sum of abortions reported by known and unknown ethnicity.

^¶^ Because reporting is not mandatory, information could not be obtained for all abortions performed in the District of Columbia.

** Data from hospitals and licensed ambulatory care facilities only; because reporting is not mandatory for private physicians and women's centers, information could not be obtained for all abortions performed in New Jersey.

^††^ Non-Hispanic category includes abortions for women whose ethnicity was reported as unknown; previous evaluation has shown that most reports without ethnicity are for non-Hispanic women.

^§§^ Percentage is based on a total of 419,065 abortions reported among the areas that met reporting standards for ethnicity.

^¶¶^ Number of abortions obtained by women in a given ethnic group per 1,000 women in that same ethnic group. For each reporting area, abortions for women of unknown ethnicity were distributed according to the distribution of abortions among women of known ethnicity for that area.

*** Number of abortions obtained by women in a given ethnic group per 1,000 live births to women in that same ethnic group. For each reporting area, abortions for women of unknown ethnicity were distributed according to the distribution of abortions among women of known ethnicity for that area.

Among the 20 areas^§§§§^ that reported by race/ethnicity for 2007, 2010, 2011, 2014, and 2015, abortion rates decreased substantially for the three largest race/ethnicity groups: for non-Hispanic white women, the abortion rate decreased 30% (from 9.4 abortions per 1,000 women in 2007 to 6.6 in 2015), for non-Hispanic black women it decreased 29% (from 36.5 abortions per 1,000 women in 2007 to 25.8 in 2015), and for Hispanic women it decreased 45% (from 21.0 abortions per 1,000 women in 2007 to 11.6 in 2015). For women in the three largest race/ethnicity groups, abortion rates decreased both from 2007 to 2010 and from 2011 to 2015, although the decreases were greater during the later period. From 2007 to 2010, the abortion rates decreased 10% for non-Hispanic white women (from 9.4 to 8.5 abortions per 1,000), 4% for non-Hispanic black women (from 36.5 to 34.9 abortions per 1,000), and 10% for Hispanic women (from 21.0 to 19.0 abortions per 1,000); by contrast, from 2011 to 2015, the abortion rates decreased 19% for non-Hispanic white women (from 8.1 to 6.6 abortions per 1,000), 20% for non-Hispanic black women (from 32.3 to 25.8 abortions per 1,000), and 31% for Hispanic women (from 16.9 to 11.6 abortions per 1,000).

Abortion ratios also decreased from 2007 to 2015 for the three largest race/ethnicity groups: for non-Hispanic white women, the abortion ratio decreased 27% (from 147 abortions per 1,000 live births in 2007 to 108 in 2015), for non-Hispanic black women it decreased 22% (from 514 abortions per 1,000 live births in 2007 to 403 in 2015), and for Hispanic women it decreased 26% (from 205 abortions per 1,000 live births in 2007 to 152 in 2015). From 2007 to 2010, abortion ratios only decreased among non-Hispanic white women (6% from 147 abortion per 1,000 live births in 2007 to 138 in 2010), whereas abortion ratios increased among non-Hispanic black women (3% from 514 abortions per 1,000 live births in 2007 to 531 in 2010) and Hispanic women (8% from 205 abortion per 1,000 live births in 2007 to 222 in 2010). By contrast, from 2011 to 2015, abortion ratios decreased among all women in the three largest race/ethnicity groups. The abortion ratio decreased 18% for non-Hispanic white women (from 132 to 108 abortions per 1,000 live births), 20% for non-Hispanic black women (from 501 to 403 abortions per 1,000 live births), and 28% for Hispanic women (from 211 to 152 abortions per 1,000 live births).

### Marital Status

Among the 39 areas that reported by marital status for 2015, 14.3% of all women who obtained an abortion were married, and 85.7% were unmarried (Table 15). The abortion ratio was 41 abortions per 1,000 live births for married women and 373 abortions per 1,000 live births for unmarried women. Among the 30 reporting areas^¶¶¶¶^ that provided these data for the relevant years of comparison (2006 versus 2015, 2006 versus 2010, 2011 versus 2015, and 2014 versus 2015), the percentage of abortions among unmarried women increased 3% from 2006 to 2015 (from 83.6% to 85.9%), with a larger increase from 2006 to 2010 (2%) than from 2011 to 2015 (<1%). Among unmarried women, the abortion ratio decreased 21% from 2006 to 2015 (from 415 to 327 abortions per 1,000 live births), with a larger decrease also occurring from 2011 to 2015 (14%) than from 2006 to 2010 (6%). Among married women, the abortion ratio decreased 31% from 2006 to 2015 (from 49 to 34 abortions per 1,000 live births), with a larger decrease occurring from 2011 to 2015 (19%) than from 2006 to 2010 (10%).

**TABLE 15:** Reported abortions, by known marital status and reporting area of occurrence — selected reporting areas,* United States, 2015

State/Area	Marital status		Total abortions reported by known marital status
	Married	Unmarried	
	No. (%)^†^	No. (%)	No. (% of all reported abortions)^§^
Alabama	643 (10.9)	5,231 (89.1)	**5,874 (99.6)**
Alaska	247 (18.7)	1,075 (81.3)	**1,322 (99.1)**
Arkansas	528 (14.0)	3,234 (86.0)	**3,762 (99.8)**
Colorado	1,778 (18.4)	7,865 (81.6)	**9,643 (95.3)**
Delaware	332 (11.9)	2,453 (88.1)	**2,785 (100.0)**
Georgia	4,143 (13.8)	25,897 (86.2)	**30,040 (96.9)**
Hawaii	546 (27.2)	1,464 (72.8)	**2,010 (99.2)**
Idaho	231 (18.8)	997 (81.2)	**1,228 (96.5)**
Illinois	4,017 (11.8)	30,151 (88.2)	**34,168 (85.7)**
Indiana	986 (12.4)	6,969 (87.6)	**7,955 (100.0)**
Iowa	578 (14.5)	3,411 (85.5)	**3,989 (100.0)**
Kansas	1,063 (15.4)	5,828 (84.6)	**6,891 (99.4)**
Kentucky	511 (16.0)	2,677 (84.0)	**3,188 (100.0)**
Louisiana	1,025 (11.2)	8,113 (88.8)	**9,138 (97.6)**
Maine	262 (14.6)	1,529 (85.4)	**1,791 (97.5)**
Massachusetts	2,440 (15.0)	13,834 (85.0)	**16,274 (87.6)**
Michigan	2,910 (10.7)	24,234 (89.3)	**27,144 (100.0)**
Minnesota	1,472 (16.2)	7,640 (83.8)	**9,112 (92.4)**
Mississippi	229 (9.2)	2,263 (90.8)	**2,492 (95.4)**
Missouri	722 (15.4)	3,959 (84.6)	**4,681 (98.2)**
Montana	232 (14.4)	1,379 (85.6)	**1,611 (100.0)**
Nebraska	258 (13.1)	1,714 (86.9)	**1,972 (98.4)**
Nevada	1,048 (16.2)	5,419 (83.8)	**6,467 (90.9)**
New Jersey^¶^	2,860 (12.6)	19,815 (87.4)	**22,675 (98.6)**
New Mexico	675 (15.3)	3,749 (84.7)	**4,424 (94.8)**
New York City	8,846 (15.9)	46,849 (84.1)	**55,695 (87.5)**
North Dakota	190 (16.3)	975 (83.7)	**1,165 (99.9)**
Oklahoma	892 (18.9)	3,816 (81.1)	**4,708 (100.0)**
Oregon	1,440 (19.0)	6,143 (81.0)	**7,583 (88.1)**
Pennsylvania	3,851 (12.1)	27,923 (87.9)	**31,774 (99.9)**
South Carolina	407 (7.0)	5,368 (93.0)	**5,775 (99.9)**
South Dakota	83 (18.7)	361 (81.3)	**444 (100.0)**
Tennessee	1,312 (11.8)	9,795 (88.2)	**11,107 (97.3)**
Texas	9,139 (16.9)	44,781 (83.1)	**53,920 (100.0)**
Utah	793 (25.3)	2,339 (74.7)	**3,132 (98.6)**
Vermont	234 (19.5)	966 (80.5)	**1,200 (94.9)**
Virginia	2,670 (14.3)	15,993 (85.7)	**18,663 (100.0)**
West Virginia	256 (17.0)	1,250 (83.0)	**1,506 (99.3)**
Wisconsin	667 (11.8)	4,983 (88.2)	**5,650 (99.8)**
**Total**	**60,516 (14.3)**	**362,442 (85.7)**	422,958 (95.2)**
**Abortion ratio^††^**	**41**	**373**	**174**

* Data from 39 reporting areas; excludes 13 areas (Arizona, California, Connecticut, District of Columbia, Florida, Maryland, New Hampshire, New York State, North Carolina, Ohio, Rhode Island, Washington, and Wyoming) that did not report, did not report by marital status, or did not meet reporting standards.

^†^ Percentages for the individual component categories might not add to 100 because of rounding.

^§^ Percentage is calculated as the number of abortions reported by known marital status divided by the sum of abortions reported by known and unknown marital status.

^¶^ Data from hospitals and licensed ambulatory care facilities only; because reporting is not mandatory for private physicians and women's centers, information could not be obtained for all abortions performed in New Jersey.

** Percentage is based on a total of 444,482 abortions reported among the areas that met reporting standards for marital status.

^††^ Number of abortions obtained by women by marital status per 1,000 live births to women of the same marital status. For each reporting area, abortions for women of unknown marital status were distributed according to the distribution of abortions among women of known marital status for that area**.**

### Previous Live Births and Abortions

Data from the 40 areas that reported the number of previous live births for women who obtained abortions in 2015 indicate that 40.7%, 45.1%, and 14.2% of these women had zero, one or two, or three or more previous live births, respectively (Table 16). Among the 35 reporting areas***** that provided these data for the relevant years of comparison (2006 versus 2015, 2006 versus 2010, 2011 versus 2015, and 2014 versus 2015), the percentage of women obtaining abortions with no previous live births was stable; by contrast, the percentage decreased 3% for women who had one or two previous live births and increased 13% for women with three or more previous live births. Among the areas included in this comparison, 40.6%, 46.6%, and 12.8% of women had zero, one to two, or three or more previous live births, respectively, in 2006; by comparison 40.6%, 45.0%, and 14.4% of women had zero, one or two, or three or more previous live births, respectively, in 2015.

**TABLE 16:** Reported abortions, by known number of previous live births and reporting area of occurrence — selected reporting areas,* United States, 2015

State/Area	No. of previous live births					Total abortions reported by known number of previous live births
	0	1	2	3	≥4	
	No. (%)^†^	No. (%)	No. (%)	No. (%)	No. (%)	No. (% of all reported abortions)^§^
Alabama	2,198 (37.3)	1,658 (28.1)	1,232 (20.9)	523 (8.9)	286 (4.8)	**5,897 (100.0)**
Alaska	597 (45.4)	292 (22.2)	217 (16.5)	103 (7.8)	107 (8.1)	**1,316 (98.7)**
Arizona	5,358 (42.4)	2,928 (23.2)	2,391 (18.9)	1,141 (9.0)	811 (6.4)	**12,629 (99.8)**
Arkansas	1,251 (33.2)	1,110 (29.5)	805 (21.4)	393 (10.4)	209 (5.5)	**3,768 (99.9)**
Colorado	5,362 (53.7)	1,946 (19.5)	1,551 (15.5)	728 (7.3)	405 (4.1)	**9,992 (98.8)**
Delaware	1,098 (39.4)	752 (27.0)	548 (19.7)	234 (8.4)	153 (5.5)	**2,785 (100.0)**
Georgia	12,275 (39.8)	7,769 (25.2)	6,073 (19.7)	2,824 (9.2)	1,875 (6.1)	**30,816 (99.4)**
Idaho	582 (45.8)	292 (23.0)	218 (17.2)	107 (8.4)	71 (5.6)	**1,270 (99.8)**
Indiana	2,943 (37.0)	2,067 (26.0)	1,693 (21.3)	783 (9.8)	471 (5.9)	**7,957 (100.0)**
Iowa	1,694 (42.5)	901 (22.6)	781 (19.6)	382 (9.6)	231 (5.8)	**3,989 (100.0)**
Kansas	2,733 (39.4)	1,720 (24.8)	1,396 (20.1)	735 (10.6)	347 (5.0)	**6,931 (100.0)**
Kentucky	1,254 (39.3)	894 (28.0)	625 (19.6)	287 (9.0)	128 (4.0)	**3,188 (100.0)**
Louisiana	3,037 (32.5)	2,639 (28.2)	2,216 (23.7)	941 (10.1)	525 (5.6)	**9,358 (100.0)**
Maine	928 (50.6)	405 (22.1)	321 (17.5)	119 (6.5)	62 (3.4)	**1,835 (99.9)**
Massachusetts	7,496 (47.3)	3,821 (24.1)	2,835 (17.9)	1,131 (7.1)	552 (3.5)	**15,835 (85.3)**
Michigan^¶^	9,641 (35.5)	7,420 (27.3)	5,675 (20.9)	2,603 (9.6)	1,804 (6.6)	**27,143 (100.0)**
Minnesota	3,942 (40.0)	2,305 (23.4)	2,035 (20.6)	901 (9.1)	678 (6.9)	**9,861 (100.0)**
Mississippi	802 (30.7)	759 (29.0)	623 (23.8)	282 (10.8)	147 (5.6)	**2,613 (100.0)**
Missouri	1,833 (38.8)	1,267 (26.8)	924 (19.5)	457 (9.7)	247 (5.2)	**4,728 (99.2)**
Montana	820 (50.9)	355 (22.0)	249 (15.5)	126 (7.8)	61 (3.8)	**1,611 (100.0)**
Nebraska	768 (38.4)	432 (21.6)	422 (21.1)	238 (11.9)	142 (7.1)	**2,002 (99.9)**
Nevada	3,026 (42.5)	1,621 (22.8)	1,357 (19.1)	672 (9.4)	438 (6.2)	**7,114 (100.0)**
New Jersey**	9,521 (41.5)	6,409 (27.9)	4,049 (17.6)	1,802 (7.8)	1,187 (5.2)	**22,968 (99.9)**
New Mexico	1,651 (38.8)	1,098 (25.8)	804 (18.9)	403 (9.5)	300 (7.0)	**4,256 (91.2)**
New York City	26,608 (44.0)	15,469 (25.6)	11,186 (18.5)	4,500 (7.4)	2,672 (4.4)	**60,435 (95.0)**
North Dakota	473 (40.6)	275 (23.6)	229 (19.6)	111 (9.5)	78 (6.7)	**1,166 (100.0)**
Ohio	7,694 (36.9)	5,532 (26.6)	4,308 (20.7)	2,028 (9.7)	1,264 (6.1)	**20,826 (99.3)**
Oklahoma	1,891 (40.2)	1,198 (25.5)	950 (20.2)	427 (9.1)	239 (5.1)	**4,705 (99.9)**
Oregon	4,202 (49.4)	1,893 (22.3)	1,415 (16.6)	625 (7.4)	367 (4.3)	**8,502 (98.7)**
Pennsylvania	12,330 (38.8)	8,592 (27.0)	6,466 (20.3)	2,800 (8.8)	1,630 (5.1)	**31,818 (100.0)**
Rhode Island	1,175 (45.0)	666 (25.5)	478 (18.3)	204 (7.8)	91 (3.5)	**2,614 (98.7)**
South Carolina	2,444 (42.3)	1,491 (25.8)	1,172 (20.3)	435 (7.5)	236 (4.1)	**5,778 (100.0)**
South Dakota	168 (37.8)	105 (23.6)	97 (21.8)	51 (11.5)	23 (5.2)	**444 (100.0)**
Tennessee	3,855 (35.2)	2,744 (25.0)	2,282 (20.8)	1,229 (11.2)	847 (7.7)	**10,957 (96.0)**
Texas	20,457 (37.9)	13,495 (25.0)	11,427 (21.2)	5,376 (10.0)	3,179 (5.9)	**53,934 (100.0)**
Utah	1,605 (51.0)	595 (18.9)	529 (16.8)	255 (8.1)	162 (5.1)	**3,146 (99.1)**
Vermont	627 (50.2)	272 (21.8)	224 (17.9)	88 (7.0)	38 (3.0)	**1,249 (98.7)**
Virginia	7,460 (40.0)	4,832 (25.9)	3,889 (20.8)	1,596 (8.6)	886 (4.7)	**18,663 (100.0)**
Washington	7,988 (46.7)	3,899 (22.8)	3,084 (18.0)	1,328 (7.8)	790 (4.6)	**17,089 (99.9)**
West Virginia	436 (28.8)	472 (31.1)	356 (23.5)	183 (12.1)	69 (4.6)	**1,516 (100.0)**
**Total**	**180,223 (40.7)**	**112,390 (25.4)**	**87,132 (19.7)**	**39,151 (8.8)**	**23,808 (5.4)**	**442,704** **(98.3)**^††^

* Data from 40 reporting areas; excludes 12 areas (California, Connecticut, District of Columbia, Florida, Hawaii, Illinois, Maryland, New Hampshire, New York State, North Carolina, Wisconsin, and Wyoming) that did not report, did not report by number of previous births, or did not meet reporting standards.

^†^ Percentages for the individual component categories might not add to 100 because of rounding.

^§^ Percentage is calculated as the number of abortions reported by known number of previous live births, divided by the sum of abortions reported by known and unknown number of previous live births.

^¶^ Recorded as the number of previous pregnancies carried to term.

** Data from hospitals and licensed ambulatory care facilities only; because reporting is not mandatory for private physicians and women's centers, information could not be obtained for all abortions performed in New Jersey.

^††^ Percentage is based on a total of 450,318 abortions reported among the areas that met reporting standards for the number of previous births.

Data from the 39 areas that reported the number of previous abortions for women who obtained abortions in 2015 indicate that the majority (56.3%) had no previous abortions, 35.4% had one or two previous abortions, and 8.2% had three or more previous abortions (Table 17). Among the 35 reporting areas^†††††^ that provided data for the relevant years of comparison (2006 versus 2015, 2006 versus 2010, 2011 versus 2015, and 2014 versus 2015), the percentage of women who had no previous abortions increased minimally (from 55.6% to 56.0%), whereas there was a 3% decrease for women who had one or two previous abortions and a 9% increase for women who had three or more previous abortions from 2006 to 2015. However, the percentage of women who had no previous abortions decreased 1% from 2006 to 2010 (from 55.6% to 54.8%) and then increased 4% from 2011 to 2015 (from 53.8% to 56.0%). By contrast, the percentage of women who had three or more previous abortions increased 12% from 2006 to 2010 (from 7.8% to 8.7%) then decreased 9% from 2011 to 2015 (from 9.3% to 8.5%). The percentage of women who had one or two previous abortions remained stable from 2006 to 2010 (36.6% to 36.5%) and then decreased 4% from 2011 to 2015 (from 36.9% to 35.5%).

**TABLE 17:** Reported abortions, by known number of previous induced abortions and reporting area of occurrence — selected reporting areas,* United States, 2015

State/Area	Number of previous induced abortions				Total abortions reported by known no. of previous induced abortions
	0	1	2	≥3	
	No. (%)^†^	No. (%)	No. (%)	No. (%)	No. (% of all reported abortions)^§^
Alabama	3,890 (66.0)	1,440 (24.4)	401 (6.8)	166 (2.8)	**5,897 (100.0)**
Alaska	886 (66.6)	291 (21.9)	100 (7.5)	54 (4.1)	**1,331 (99.8)**
Arizona	7,958 (63.3)	3,063 (24.4)	1,033 (8.2)	513 (4.1)	**12,567 (99.3)**
Arkansas	2,219 (58.9)	845 (22.4)	360 (9.6)	344 (9.1)	**3,768 (99.9)**
Colorado	6,561 (65.4)	2,409 (24.0)	743 (7.4)	312 (3.1)	**10,025 (99.1)**
Delaware	1,633 (58.6)	668 (24.0)	290 (10.4)	194 (7.0)	**2,785 (100.0)**
Georgia	19,033 (61.7)	7,327 (23.7)	2,957 (9.6)	1,547 (5.0)	**30,864 (99.5)**
Idaho	932 (73.4)	252 (19.9)	59 (4.6)	26 (2.0)	**1,269 (99.8)**
Indiana	5,087 (63.9)	1,943 (24.4)	635 (8.0)	292 (3.7)	**7,957 (100.0)**
Iowa	2,550 (64.0)	964 (24.2)	306 (7.7)	163 (4.1)	**3,983 (99.8)**
Kansas	4,489 (64.8)	1,613 (23.3)	547 (7.9)	282 (4.1)	**6,931 (100.0)**
Kentucky	2,032 (65.3)	733 (23.6)	258 (8.3)	88 (2.8)	**3,111 (97.6)**
Louisiana	5,616 (60.0)	2,509 (26.8)	851 (9.1)	382 (4.1)	**9,358 (100.0)**
Maine	1,186 (65.4)	443 (24.4)	136 (7.5)	49 (2.7)	**1,814 (98.8)**
Massachusetts	9,231 (52.6)	4,575 (26.1)	2,082 (11.9)	1,673 (9.5)	**17,561 (94.6)**
Michigan	13,637 (50.2)	6,884 (25.4)	3,789 (14.0)	2,837 (10.5)	**27,147 (100.0)**
Minnesota	5,902 (59.9)	2,308 (23.4)	948 (9.6)	703 (7.1)	**9,861 (100.0)**
Mississippi	1,709 (65.4)	600 (23.0)	223 (8.5)	81 (3.1)	**2,613 (100.0)**
Missouri	2,996 (62.9)	1,170 (24.6)	428 (9.0)	170 (3.6)	**4,764 (100.0)**
Montana	563 (34.9)	716 (44.4)	221 (13.7)	111 (6.9)	**1,611 (100.0)**
Nebraska	1,250 (62.4)	503 (25.1)	182 (9.1)	69 (3.4)	**2,004 (100.0)**
Nevada	3,853 (54.1)	1,890 (26.6)	767 (10.8)	606 (8.5)	**7,116 (100.0)**
New Jersey^¶^	15,297 (66.6)	3,889 (16.9)	1,914 (8.3)	1,870 (8.1)	**22,970 (99.9)**
New York City	23,240 (38.9)	13,775 (23.0)	11,021 (18.4)	11,730 (19.6)	**59,766 (93.9)**
North Dakota	753 (64.6)	273 (23.4)	85 (7.3)	55 (4.7)	**1,166 (100.0)**
Ohio	12,073 (58.2)	5,427 (26.2)	2,042 (9.8)	1,193 (5.8)	**20,735 (98.9)**
Oklahoma	3,143 (66.8)	1,053 (22.4)	325 (6.9)	181 (3.8)	**4,702 (99.9)**
Oregon	5,049 (59.3)	2,077 (24.4)	844 (9.9)	547 (6.4)	**8,517 (98.9)**
Pennsylvania	16,826 (52.9)	8,041 (25.3)	3,889 (12.2)	3,062 (9.6)	**31,818 (100.0)**
Rhode Island	1,392 (54.3)	691 (27.0)	262 (10.2)	218 (8.5)	**2,563 (96.8)**
South Carolina	3,273 (56.6)	1,381 (23.9)	661 (11.4)	463 (8.0)	**5,778 (100.0)**
South Dakota	300 (67.6)	88 (19.8)	31 (7.0)	25 (5.6)	**444 (100.0)**
Tennessee	5,837 (53.2)	2,921 (26.6)	1,319 (12.0)	903 (8.2)	**10,980 (96.2)**
Texas	32,524 (60.3)	14,031 (26.0)	4,932 (9.1)	2,438 (4.5)	**53,925 (100.0)**
Utah	2,467 (77.7)	504 (15.9)	115 (3.6)	90 (2.8)	**3,176 (100.0)**
Vermont	772 (61.4)	294 (23.4)	123 (9.8)	68 (5.4)	**1,257 (99.4)**
Virginia	10,697 (57.3)	4,681 (25.1)	2,066 (11.1)	1,219 (6.5)	**18,663 (100.0)**
Washington	9,766 (57.2)	4,102 (24.0)	1,809 (10.6)	1,406 (8.2)	**17,083 (99.9)**
West Virginia	781 (51.5)	426 (28.1)	190 (12.5)	119 (7.8)	**1,516 (100.0)**
**Total**	**247,403 (56.3)**	**106,800 (24.3)**	**48,944 (11.1)**	**36,249 (8.2)**	439,396 (98.6)**

* Data from 39 reporting areas; excludes 13 areas (California, Connecticut, District of Columbia, Florida, Hawaii, Illinois, Maryland, New Hampshire, New Mexico, New York State, North Carolina, Wisconsin, and Wyoming) that did not report, did not report by the number of previous induced abortions, or did not meet reporting standards.

^†^ Percentages for the individual component categories might not add to 100 because of rounding.

^§^ Percentage is calculated as the number of abortions reported by known number of previous induced abortions divided by the sum of abortions reported by known and unknown number of previous induced abortions.

^¶^ Data from hospitals and licensed ambulatory care facilities only; because reporting is not mandatory for private physicians and women's centers, information could not be obtained for all abortions performed in New Jersey.

** Percentage is based on a total of 445,649 abortions reported among the areas that met reporting standards for the number of previous abortions.

### Maternal Age and Marital Status by Race/Ethnicity

In select reporting areas, abortions that were categorized by maternal race and race/ethnicity were further categorized by maternal age and by marital status (Tables 18 and 19). A consistent pattern existed for abortions by maternal age across all race/ethnicity groups, with the smallest percentage of abortions occurring among adolescents aged <15 years (0.2%–0.3%) and the largest percentage occurring among women aged 20–24 years (26.5%–32.2%) and 25–29 years (26.7%–28.8%) (Table 19). A consistent pattern also existed for abortions by marital status across all race/ethnicity groups, with a higher percentage of abortions occurring among women who were unmarried (69.0%–91.8%) than among those who were married (8.2%–31.0%) (Table 19). For abortions among married women, the percentage was higher for non-Hispanic women in the other race group (31.0%) than for non-Hispanic white women (17.0%), Hispanic (15.6%) women, or non-Hispanic black women (8.2%). For abortions among unmarried women, the percentage was higher for non-Hispanic black women (91.8%) than for non-Hispanic white (83.0%) women, Hispanic (84.4%) women, or non-Hispanic women in the other race group (69.0%) (Table 19).

**TABLE 18:** Reported abortions, by known race, age group, and marital status of women who obtained an abortion — selected reporting areas, United States, 2015

Characteristic	Race			Total
	White	Black	Other	
	No. (%)*	No. (%)	No. (%)	No. (%)
**Age group (yrs)^†^**				
<15	277 (0.2)	350 (0.3)	65 (0.2)	**692 (0.3)**
15–19	12,337 (10.1)	10,011 (9.4)	2,421 (9.1)	**24,769 (9.7)**
15	617 (0.5)	654 (0.6)	113 (0.4)	**1,384 (0.5)**
16	1,061 (0.9)	1,041 (1.0)	239 (0.9)	**2,341 (0.9)**
17	1,824 (1.5)	1,542 (1.5)	352 (1.3)	**3,718 (1.5)**
18	3,699 (3.0)	2,828 (2.7)	707 (2.7)	**7,234 (2.8)**
19	5,136 (4.2)	3,946 (3.7)	1,010 (3.8)	**10,092 (4.0)**
20–24	38,349 (31.3)	35,152 (33.1)	7,671 (28.8)	**81,172 (31.8)**
25–29	32,849 (26.8)	30,939 (29.1)	6,923 (26.0)	**70,711 (27.7)**
30–34	21,404 (17.5)	17,936 (16.9)	5,013 (18.9)	**44,353 (17.4)**
35–39	12,607 (10.3)	9,256 (8.7)	3,182 (12.0)	**25,045 (9.8)**
≥40	4,695 (3.8)	2,596 (2.4)	1,315 (4.9)	**8,606 (3.4)**
**Total**	**122,518 (100.0)**	**106,240 (100.0)**	**26,590 (100.0)**	**255,348 (100.0)**
**Marital status^§^**				
Married	15,934 (16.1)	5,977 (7.3)	6,026 (25.8)	**27,937 (13.7)**
Unmarried	82,847 (83.9)	76,313 (92.7)	17,364 (74.2)	**176,524 (86.3)**
**Total**	**98,781 (100.0)**	**82,290 (100.0)**	**23,390 (100.0)**	**204,461 (100.0)**

* Percentages for the individual component categories might not add to 100 because of rounding.

^†^ Data from 34 reporting areas; excludes 18 areas (Arizona, California, Connecticut, Florida, Illinois, Kentucky, Maryland, Massachusetts, Nevada, New Hampshire, New Mexico, New York State, New York City, Pennsylvania, Texas, Washington, Wisconsin, and Wyoming) that did not report, did not report race by age, or did not meet reporting standards.

^§^ Data from 30 reporting areas; excludes 22 areas (Arizona, California, Connecticut, District of Columbia, Florida, Illinois, Kentucky, Maryland, Massachusetts, Nevada, New Hampshire, New Mexico, New York State, New York City, North Carolina, Ohio, Pennsylvania, Rhode Island, Texas, Washington, Wisconsin, and Wyoming) that did not report, did not report race by marital status, or did not meet reporting standards.

**TABLE 19:** Reported abortions, by known race/ethnicity, age group, and marital status of women who obtained an abortion — selected reporting areas, United States, 2015

Characteristic	Non-Hispanic			Hispanic	Total
	White	Black	Other		
	No. (%)*	No. (%)	No. (%)	No. (%)	No. (%)
**Age group (yrs)^†^**					
<15	238 (0.2)	369 (0.3)	50 (0.2)	218 (0.3)	**875 (0.3)**
15–19	11,683 (9.5)	11,501 (9.5)	2,215 (7.6)	6,541 (10.5)	**31,940 (9.5)**
15	551 (0.4)	709 (0.6)	103 (0.4)	339 (0.5)	**1,702 (0.5)**
16	935 (0.8)	1,168 (1.0)	193 (0.7)	660 (1.1)	**2,956 (0.9)**
17	1,716 (1.4)	1,830 (1.5)	308 (1.1)	1,003 (1.6)	**4,857 (1.4)**
18	3,485 (2.8)	3,253 (2.7)	654 (2.2)	1,878 (3.0)	**9,270 (2.8)**
19	4,996 (4.1)	4,541 (3.8)	957 (3.3)	2,661 (4.3)	**13,155 (3.9)**
20–24	37,275 (30.4)	38,876 (32.2)	7,724 (26.5)	19,939 (31.9)	**103,814 (31.0)**
25–29	33,410 (27.2)	34,770 (28.8)	7,774 (26.7)	16,703 (26.7)	**92,657 (27.7)**
30–34	21,943 (17.9)	20,915 (17.3)	5,829 (20.0)	10,840 (17.3)	**59,527 (17.8)**
35–39	13,104 (10.7)	11,022 (9.1)	3,822 (13.1)	6,122 (9.8)	**34,070 (10.2)**
≥40	4,955 (4.0)	3,363 (2.8)	1,684 (5.8)	2,122 (3.4)	**12,124 (3.6)**
**Total**	**122,608 (100.0)**	**120,816 (100.0)**	**29,098 (100.0)**	**62,485 (100.0)**	**335,007 (100.0)**
**Marital status^§^**					
Married	17,558 (17.0)	8,840 (8.2)	8,110 (31.0)	8,708 (15.6)	**43,216 (14.7)**
Unmarried	85,900 (83.0)	98,886 (91.8)	18,065 (69.0)	47,021 (84.4)	**249,872 (85.3)**
**Total**	**103,458 (100.0)**	**107,726 (100.0)**	**26,175 (100.0)**	**55,729 (100.0)**	**293,088 (100.0)**

* Percentages for the individual component categories might not add to 100 because of rounding.

^†^ Data from 29 reporting areas; excludes 23 areas (California, Connecticut, Florida, Illinois, Iowa, Kentucky, Louisiana, Maine, Maryland, Massachusetts, Mississippi, Nebraska, New Hampshire, New Mexico, New York State, North Carolina, North Dakota, Oklahoma, Pennsylvania, Rhode Island, Washington, Wisconsin, and Wyoming) that did not report, did not report race/ethnicity by age, or did not meet reporting standards.

^§^ Data from 26 reporting areas; excludes 26 reporting areas (Arizona, California, Connecticut, District of Columbia, Florida, Illinois, Iowa, Kentucky, Louisiana, Maine, Maryland, Massachusetts, Mississippi, Nebraska, New Hampshire, New Mexico, New York State, North Carolina, North Dakota, Ohio, Oklahoma, Pennsylvania, Rhode Island, Washington, Wisconsin, and Wyoming) that did not report, did not report race/ethnicity by marital status, or did not meet reporting standards.

### Weeks of Gestation by Maternal Age, Race/Ethnicity, and Method Type

**FIGURE 3:**
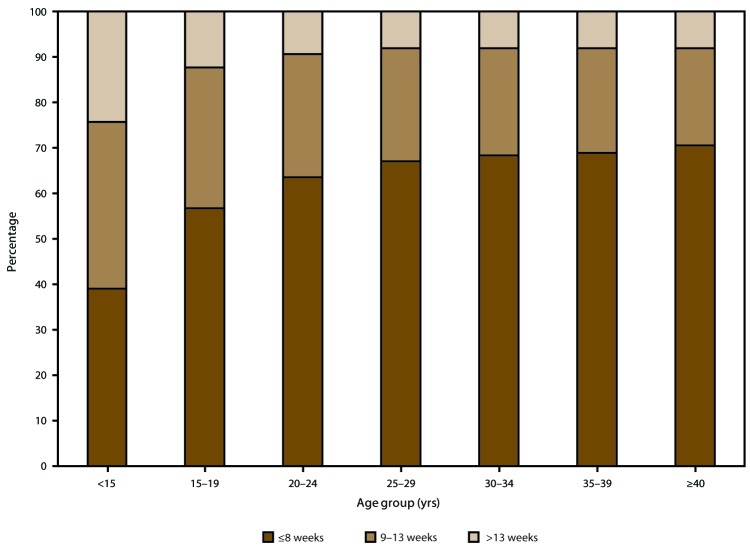
Percentage* distribution of gestational ages at time of abortion, by age of woman — selected reporting areas,^†^ United States, 2015 * Based on the total number of abortions reported with known weeks of gestation. ^†^ Data from 39 reporting areas; excludes 13 reporting areas (California, Connecticut, District of Columbia, Florida, Illinois, Kentucky, Maryland, Massachusetts, New Hampshire, New York State, Pennsylvania, Wisconsin, and Wyoming) that did not report, did not report by weeks of gestation by age, or did not meet reporting standards.

In certain reporting areas, abortions that were categorized by weeks of gestation were further categorized by maternal age and race/ethnicity (Tables 20 and 21). In every subgroup for these three variables, the largest percentage of abortions occurred at ≤8 weeks’ gestation. However, by maternal age, 39.0% of adolescents aged <15 years and 56.7% of adolescents aged 15–19 years obtained an abortion by ≤8 weeks’ gestation, compared with 63.5%–70.5% of women in older age groups (Figure 3) (Table 20). Conversely, 24.3% of adolescents aged <15 years and 12.3% of adolescents aged 15–19 years obtained an abortion after 13 weeks’ gestation, compared with 8.0%–9.4% for women in older age groups. By race/ethnicity, 59.1% of non-Hispanic black women obtained an abortion at ≤8 weeks’ gestation, compared with 67.5%–70.3% of women from other race/ethnicity groups. Differences in abortions after 13 weeks’ gestation across race/ethnicity groups were less apparent than differences across age groups (10.5% for non-Hispanic black women, compared with 8.0%–8.5% for women in the remaining race/ethnicity groups).

**TABLE 20:** Reported abortions, by known weeks of gestation, age group, and race/ethnicity of women who obtained an abortion — selected reporting areas, United States, 2015

Characteristic	Weeks of gestation					
	≤8	9–13	14–15	16–17	18–20	≥21
	No. (%)	No. (%)	No. (%)	No. (%)	No. (%)	No. (%)
Age group (yrs)*^,†^						
<15	441 (39.0)	416 (36.8)	81 (7.2)	72 (6.4)	55 (4.9)	66 (5.8)
15–19	23,096 (56.7)	12,644 (31.0)	1,855 (4.6)	1,155 (2.8)	1,133 (2.8)	841 (2.1)
20–24	83,216 (63.5)	35,597 (27.2)	4,950 (3.8)	2,956 (2.3)	2,625 (2.0)	1,655 (1.3)
25–29	77,945 (67.0)	28,969 (24.9)	3,889 (3.3)	2,223 (1.9)	2,010 (1.7)	1,318 (1.1)
30–34	51,351 (68.4)	17,720 (23.6)	2,322 (3.1)	1,363 (1.8)	1,368 (1.8)	951 (1.3)
35–39	29,596 (68.9)	9,863 (23.0)	1,374 (3.2)	817 (1.9)	792 (1.8)	502 (1.2)
≥40	10,790 (70.5)	3,285 (21.5)	460 (3.0)	282 (1.8)	290 (1.9)	200 (1.3)
**Total**	**276,435 (65.4)**	**108,494 (25.7)**	**14,931 (3.5)**	**8,868 (2.1)**	**8,273 (2.0)**	**5,533 (1.3)**
Race/Ethnicity*^,§^						
Non-Hispanic						
White	82,889 (67.7)	29,683 (24.2)	3,898 (3.2)	2,213 (1.8)	2,249 (1.8)	1,510 (1.2)
Black	70,937 (59.1)	36,360 (30.3)	4,931 (4.1)	3,034 (2.5)	2,909 (2.4)	1,790 (1.5)
Other	20,448 (70.3)	6,252 (21.5)	900 (3.1)	584 (2.0)	580 (2.0)	343 (1.2)
Hispanic	42,110 (67.5)	14,970 (24.0)	2,161 (3.5)	1,248 (2.0)	1,186 (1.9)	703 (1.1)
**Total**	**216,384 (64.8)**	**87,265 (26.1)**	**11,890 (3.6)**	**7,079 (2.1)**	**6,924 (2.1)**	**4,346 (1.3)**

* Row percentages might not add to 100 because of rounding.

^†^ Data from 39 reporting areas; excludes 13 reporting areas (California, Connecticut, District of Columbia, Florida, Illinois, Kentucky, Maryland, Massachusetts, New Hampshire, New York State, Pennsylvania, Wisconsin, and Wyoming) that did not report, did not report weeks of gestation by age, or did not meet reporting standards.

^§^ Data from 28 reporting areas; excludes 24 reporting areas (California, Connecticut, District of Columbia, Florida, Illinois, Iowa, Kentucky, Louisiana, Maine, Maryland, Massachusetts, Mississippi, Nebraska, New Hampshire, New Mexico, New York State, North Carolina, North Dakota, Oklahoma, Pennsylvania, Rhode Island, Washington, Wisconsin, and Wyoming) that did not report, did not report weeks of gestation by race/ethnicity, or did not meet reporting standards.

**TABLE 21:** Reported abortions obtained at ≤13 weeks’ gestation, by known weeks of gestation, age group, and race/ethnicity of women who obtained an abortion — selected reporting areas, United States, 2015

Characteristic	Weeks of gestation							
	≤6	7	8	9	10	11	12	13
	No. (%)	No. (%)	No. (%)	No. (%)	No. (%)	No. (%)	No. (%)	No. (%)
Age group (yrs)*^,†^								
<15	189 (22.1)	133 (15.5)	119 (13.9)	118 (13.8)	85 (9.9)	87 (10.2)	66 (7.7)	60 (7.0)
15–19	11,067 (31.0)	6,489 (18.2)	5,540 (15.5)	4,119 (11.5)	2,787 (7.8)	2,422 (6.8)	1,801 (5.0)	1,515 (4.2)
20–24	42,051 (35.4)	23,164 (19.5)	18,001 (15.2)	12,059 (10.1)	8,126 (6.8)	6,691 (5.6)	4,868 (4.1)	3,853 (3.2)
25–29	41,359 (38.7)	21,159 (19.8)	15,427 (14.4)	10,300 (9.6)	6,506 (6.1)	5,274 (4.9)	3,814 (3.6)	3,075 (2.9)
30–34	27,808 (40.3)	13,751 (19.9)	9,792 (14.2)	6,357 (9.2)	3,962 (5.7)	3,196 (4.6)	2,343 (3.4)	1,862 (2.7)
35–39	16,135 (40.9)	7,883 (20.0)	5,578 (14.1)	3,644 (9.2)	2,201 (5.6)	1,622 (4.1)	1,341 (3.4)	1,055 (2.7)
≥40	6,263 (44.5)	2,710 (19.3)	1,817 (12.9)	1,180 (8.4)	684 (4.9)	599 (4.3)	437 (3.1)	385 (2.7)
**Total**	**144,872 (37.6)**	**75,289 (19.6)**	**56,274 (14.6)**	**37,777 (9.8)**	**24,351 (6.3)**	**19,891 (5.2)**	**14,670 (3.8)**	**11,805 (3.1)**
Race/Ethnicity*^,§^								
Non-Hispanic								
White	44,697 (39.7)	22,011 (19.6)	16,181 (14.4)	10,749 (9.5)	6,541 (5.8)	5,357 (4.8)	3,816 (3.4)	3,220 (2.9)
Black	33,129 (30.9)	21,052 (19.6)	16,756 (15.6)	12,047 (11.2)	8,329 (7.8)	6,940 (6.5)	5,210 (4.9)	3,834 (3.6)
Other	11,554 (43.3)	5,201 (19.5)	3,693 (13.8)	2,324 (8.7)	1,370 (5.1)	1,040 (3.9)	791 (3.0)	727 (2.7)
Hispanic	23,271 (40.8)	10,988 (19.3)	7,851 (13.8)	5,357 (9.4)	3,495 (6.1)	2,666 (4.7)	1,910 (3.3)	1,542 (2.7)
**Total**	**112,651 (37.1)**	**59,252 (19.5)**	**44,481 (14.6)**	**30,477 (10.0)**	**19,735 (6.5)**	**16,003 (5.3)**	**11,727 (3.9)**	**9,323 (3.1)**

* Row percentages might not add to 100 because of rounding.

^†^ Data from 39 reporting areas; excludes 13 reporting areas (California, Connecticut, District of Columbia, Florida, Illinois, Kentucky, Maryland, Massachusetts, New Hampshire, New York State, Pennsylvania, Wisconsin, and Wyoming) that did not report, did not report weeks of gestation by age, or did not meet reporting standards.

^§^ Data from 28 reporting areas; excludes 24 reporting areas (California, Connecticut, District of Columbia, Florida, Illinois, Iowa, Kentucky, Louisiana, Maine, Maryland, Massachusetts, Mississippi, Nebraska, New Hampshire, New Mexico, New York State, North Carolina, North Dakota, Oklahoma, Pennsylvania, Rhode Island, Washington, Wisconsin, and Wyoming) that did not report, did not report weeks of gestation by race/ethnicity, or did not meet reporting standards.

Among abortions categorized by weeks of gestation and method type, surgical abortion accounted for the largest percentage of abortions within every gestational age category (Table 22). At ≤8 weeks’ gestation, surgical abortion accounted for a smaller percentage of abortions (64.2%) than at any other stage of gestation; at 9–20 weeks’ gestation, surgical abortion accounted for 94.5%–99.2% of all abortions and at ≥21 weeks’ gestation, it accounted for 94.5% of abortions. By contrast, at ≤8 weeks’ gestation, medical abortion accounted for 35.8% of abortions then decreased to 5.5% at 9–13 weeks and 0.7%–1.9% at 14–20 weeks before increasing to 4.5% at ≥21 weeks. Throughout gestation, abortions performed by intrauterine instillation or hysterectomy/hysterotomy were rare (<0.1%–0.7% of abortions).

**TABLE 22:** Reported abortions, by known weeks of gestation and method type — selected reporting areas,* United States, 2015

Method type	Weeks of gestation						Total
	≤8	9–13	14–15	16–17	18–20	≥21	
	No. (%)^†^	No. (%)	No. (%)	No. (%)	No. (%)	No. (%)	No. (%)
**Surgical^§^**							
≤13 weeks’ gestation	166,476 (64.2)	95,604 (94.5)	NA	NA	NA	NA	**262,080 (66.2)**
>13 weeks’ gestation	NA	NA	13,768 (99.2)	8,378 (98.9)	7,735 (97.7)	4,573 (94.5)	**34,454 (8.7)**
**Medical^¶^**							
≤8 weeks’ gestation	92,971 (35.8)	NA	NA	NA	NA	NA	**92,971 (23.5)**
>8 weeks’ gestation	NA	5,537 (5.5)	91 (0.7)	82 (1.0)	153 (1.9)	219 (4.5)	**6,082 (1.5)**
**Intrauterine instillation**	—**	—^††^	13 (0.1)	—^††^	16 (0.2)	33 (0.7)	**74 (0.0)**
**Hysterectomy/Hysterotomy**	9 (0.0)	—^††^	7 (0.1)	—^††^	16 (0.2)	12 (0.2)	**55 (0.0)**
**Total**	**259,456 (100.0)**	**101,152 (100.0)**	**13,879 (100.0)**	**8,472 (100.0)**	**7,920 (100.0)**	**4,837 (100.0)**	**395,716 (100.0)**

**Abbreviation:** NA = not applicable.

* Data from 35 reporting areas; excludes 17 areas (California, Connecticut, District of Columbia, Florida, Hawaii, Illinois, Kentucky, Louisiana, Maryland, Massachusetts, New Hampshire, New Mexico, New York State, Pennsylvania, Tennessee, Wisconsin, and Wyoming) that did not report, did not report method type by weeks of gestation, did not meet reporting standards, or did not have medical abortion as a specific category on their reporting form.

^†^ For each gestational age category, percentages of all method types might not add to 100 because of rounding.

^§^ Includes aspiration curettage, suction curettage, manual vacuum aspiration, menstrual extraction, sharp curettage, and dilation and evacuation procedures.

^¶^ The administration of medication or medications to induce an abortion; at ≤8 weeks’ gestation, typically involves the use of mifepristone and misoprostol; at >8 weeks’ gestation, typically involves the use of vaginal prostaglandins.

** Intrauterine instillations reported at ≤12 weeks’ gestation have not been included with known values.

^††^ Cells with a value in the range of 1–4 or cells that would allow for calculation of these small values have been suppressed.

### Abortion Mortality

Using national data from the Pregnancy Mortality Surveillance System (*51*), CDC identified six abortion-related deaths for 2014 (Table 23). Investigation of these cases indicated that all six deaths were related to legal abortion and none to illegal abortion.

**TABLE 23:** Number of deaths and case-fatality rates* for abortion-related deaths reported to CDC, by type of abortion — United States, 1973–2014^†^

Year	Type of abortion				CFR per 100,000 legal abortions
	Induced		Unknown**	Total	
	Legal^§^	Illegal^¶^			
**1973–1977**					**2.09**
1973	25	19	3	**47**	
1974	26	6	1	**33**	
1975	29	4	1	**34**	
1976	11	2	1	**14**	
1977	17	4	0	**21**	
**1978–1982**					**0.78**
1978	9	7	0	**16**	
1979	22	0	0	**22**	
1980	9	1	2	**12**	
1981	8	1	0	**9**	
1982	11	1	0	**12**	
**1983–1987**					**0.66**
1983	11	1	0	**12**	
1984	12	0	0	**12**	
1985	11	1	1	**13**	
1986	11	0	2	**13**	
1987	7	2	0	**9**	
**1988–1992**					**0.74**
1988	16	0	0	**16**	
1989	12	1	0	**13**	
1990	9	0	0	**9**	
1991	11	1	0	**12**	
1992	10	0	0	**10**	
**1993–1997**					**0.52**
1993	6	1	2	**9**	
1994	10	2	0	**12**	
1995	4	0	0	**4**	
1996	9	0	0	**9**	
1997	7	0	0	**7**	
**1998–2002**					**0.63**
1998	9	0	0	**9**	
1999	4	0	0	**4**	
2000	11	0	0	**11**	
2001	7	1	0	**8**	
2002	10	0	0	**10**	
**2003–2007**					**0.60**
2003	10	0	0	**10**	
2004	7	1	0	**8**	
2005	7	0	0	**7**	
2006	7	0	0	**7**	
2007	6	0	0	**6**	
**2008–2014**					**0.62**
2008	12	0	0	**12**	
2009	8	0	0	**8**	
2010	10	0	0	**10**	
2011	2	0	0	**2**	
2012	4	0	0	**4**	
2013	4	0	0	**4**	
2014	6	0	0	**6**	
**Total**	**437**	**56**	**13**	**506**	**0.79**

**Abbreviation:** CFR = case-fatality rate.

* Number of legal induced abortion-related deaths per 100,000 reported legal induced abortions. Because a substantial number of legal induced abortions occurred outside reporting areas that provided data to CDC, national case-fatality rates (i.e., number of legal induced abortion-related deaths per 100,000 reported legal induced abortions in the United States) were calculated with denominator data from a more complete source *(16)*. Case-fatality rates were computed for consecutive 5-year periods during 1973–2007 and then for a consecutive 7-year period during 2008–2014 because rates based on <20 cases are highly variable *(43)* .

^†^ Certain numbers might differ from those in reports published previously because additional information has been supplied to CDC subsequent to publication.

^§^ An abortion is defined as legal if it was performed by a licensed clinician within the limits of state law.

^¶^ An abortion is defined as illegal if it was performed by any person other than a licensed clinician.

** Unknown whether abortion was induced or spontaneous.

The annual number of deaths related to legal induced abortion has fluctuated from year to year over the past 40 years (Table 23). For example, nine legal induced abortion-related deaths occurred in 1998, four in 1999, and 11 in 2000. Because of this variability and the relatively limited number of legal induced abortion-related deaths every year, national legal abortion case-fatality rates were calculated for consecutive 5-year periods during 1973–2007 and for a consecutive 7-year period during 2008–2014. The national legal induced abortion case-fatality rate for 2008–2014 was 0.62 legal induced abortion-related deaths per 100,000 reported legal abortions. This case-fatality rate was similar to the rate for most of the preceding 5-year periods but lower than the case-fatality rate of 2.09 legal induced abortion-related deaths per 100,000 reported legal abortions for the 5-year period (1973–1977) immediately following nationwide legalization of abortion in 1973. Possible abortion-related deaths that occurred during 2015–2018 are being assessed.

## Discussion

For 2015, a total of 638,169 abortions were reported to CDC by 49 areas. Among these areas, the abortion rate was 11.8 abortions per 1,000 women aged 15–44 years and the abortion ratio was 188 abortions per 1,000 live births. All 49 of these reporting areas submitted data every year during the period of analysis from 2006 to 2015, thus providing the information necessary for evaluating trends. Among these areas, the number, rate, and ratio of reported abortions decreased 2% from 2014 to 2015, which, in combination with decreases that occurred during previous years (*11*–*15*), resulted in the lowest values for all three measures for the entire period of analysis. Among areas that reported by age every year of the analysis, women in their 20s accounted for the majority of abortions (57%–59%) and had the highest abortion rates, whereas decreases in the abortion rate were greater for adolescents aged <20 years than for any other age group. In addition, throughout the period of analysis, ≤9% of abortions each year were performed after 13 weeks’ gestation; approximately two thirds of abortions were performed at ≤8 weeks’ gestation, and this percentage increased from 63.5% in 2006 to 65.4% in 2015. Among areas that included medical abortion on their reporting form every year, the percentage of all abortions performed by early medical abortion increased from 11.3% in 2006 to 24.2% in 2015.

These findings underscore important maternal age differences in abortion trends. Because of the high rate and proportion of abortions that occurred among women in their 20s, women in this age group have contributed substantially to overall changes. Conversely, during 2006–2015, women aged ≥40 years had consistently low abortion rates and accounted for a limited percentage of abortions (≤3.7%); therefore, they have had a much smaller contribution to overall abortion trends. Nonetheless, among women aged ≥40 years, the abortion ratio continues to be higher than among women in their mid to late 20s and 30s. Because of the limited proportion of abortions that are performed later in gestation among women aged ≥40 years, which might be completed for maternal medical indications or fetal anomalies, the continuing high abortion ratio among these older women suggests that unintended pregnancy is a problem that women encounter throughout their reproductive years (*52*).

The adolescent abortion trends described in this report are important for monitoring progress that has been made toward reducing adolescent pregnancies in the United States. National birth data indicate the birth rate for adolescents aged 15–19 years decreased 47% during 2006–2015 (*53*,*54*), compared with a 54% decrease in the abortion rate for adolescents aged 15–19 years during the same period. Recent national birth data indicate the birth rate decreased an additional 16% from 2015 to 2017 (*55*,*56*). These findings indicate that declines in adolescent pregnancies in the United States have been accompanied by large decreases in both adolescent births and abortions and that the pattern of decline is continuing (*53*–*56*).

The findings in this report indicate that the number, rate, and ratio of reported abortions have declined across all race/ethnicity groups but that well-documented disparities persist (*3*,*4*,*17*–*22*). In this report, abortion rates and ratios remained 1.5 and 1.3 times higher for Hispanic compared with non-Hispanic white women and 3.6 and 3.5 times higher for non-Hispanic black compared with non-Hispanic white women. The comparatively high abortion rates and ratios among non-Hispanic black women have been attributed to higher unintended pregnancy rates and a greater percentage of unintended pregnancies ending in abortion (*52*). Data from certain reports suggest that differences in abortion indicators between non-Hispanic black women and women of other groups narrowed from 1994 to 2008 (*4*,*21*), but remained steady from 2008 to 2014 (*22*).

The findings in this report indicate the majority of women obtaining abortions do so early in gestation (≤8 weeks), when the risks for complications are lowest (*57*–*60*). Among the areas that reported gestational age data every year during 2006–2015, the percentage of abortions performed at ≤8 weeks’ gestation increased 3%. Moreover, among the areas that reported abortions at ≤13 weeks’ gestation by individual week, the distribution continued to shift toward earlier weeks of gestation, with the percentage of early abortions performed at ≤6 weeks’ gestation increasing 11% from 2006 to 2015. Nonetheless, the overall percentage of abortions performed at ≤13 weeks’ gestation was stable during 2006–2015. Reports indicate that delays in obtaining an abortion are more common among certain groups of women (*61*–*63*); among women obtaining abortions in this report, a smaller percentage of adolescents aged ≤19 years and non-Hispanic black women, compared with women in other age and race/ethnicity groups, obtained abortions at ≤8 weeks’ gestation. Because of the small but persistent percentage of women who obtain abortions at >13 weeks’ gestation, a better understanding is needed of how to address delays in obtaining abortions (*61*,*63*–*66*).

The trend of obtaining abortions earlier in pregnancy has been facilitated by changes in abortion practices. Research conducted in the United States during the 1970s indicated that surgical abortion procedures performed at ≤6 weeks’ gestation, compared with 7–12 weeks’ gestation, were less likely to result in successful termination of the pregnancy (*67*). However, subsequent advances in technology (e.g., improved transvaginal ultrasonography and sensitive pregnancy tests) have allowed very early surgical abortions to be performed with completion rates exceeding 97% (*68*–*70*). Likewise, the development of early medical abortion regimens has allowed for abortions to be performed very early in gestation, with completion rates for regimens that combine mifepristone and misoprostol reaching 96%–98% (*71*). In 2015, 65.4% of all reported abortions were performed at ≤8 completed weeks’ gestation; thus, the women receiving these abortions were eligible for early medical abortion (a nonsurgical abortion at ≤8 weeks’ gestation) on the basis of gestational age; 35.8% of abortions at ≤8 weeks’ gestation and 24.6% of all abortions were reported as early medical abortions, with the proportion of all abortions reported as early medical abortion up from 11.3% in 2006. Moreover, in addition to abortions meeting the definition of early medical abortion, the percentage of abortions at 9 weeks’ gestation reported as medical has increased in recent years (from 5.0%–7.7% during 2011–2014 to 13.0% in 2015). On the basis of evidence that early medical abortion is safe and effective beyond 63 days’ gestation (*44*), professional clinical practice guidelines were updated midyear in 2013 and 2014 to extend the gestational age eligibility for early medical abortion from 63 to 70 days (≤9 completed weeks) (*45*,*46*). In early 2016, FDA updated its approval for use of mifepristone for early medical abortions, extending the gestational age limit to 70 days (*47*). CDC will continue to monitor medical abortions at 9 weeks’ gestation.

The annual number of deaths related to legal induced abortion has fluctuated from year to year over the past 40 years. Because of this variability and the relatively limited number of abortion-related deaths every year, national legal abortion case-fatality rates were calculated for consecutive 5-year periods during 1973–2007 and for a consecutive 7-year period during 2008–2014. The national legal induced abortion case-fatality rate for 2008–2014 was similar to the case-fatality rate for most of the preceding 5-year periods but was much lower than the case-fatality rate for the 5-year period (1973–1978) that immediately followed nationwide legalization of abortion in 1973.

### Limitations

The findings in this report are subject to at least four limitations. First, because reporting to CDC is voluntary and reporting requirements are established by the individual reporting areas (*24*), CDC is unable to obtain the total number of abortions performed in the United States. Although most reporting areas collect and send abortion data to CDC, three of the 52 reporting areas (California, Maryland, and New Hampshire) did not provide CDC data for 2006–2015 on a consistent annual basis. During the period covered by this report, the total annual number of abortions reported to CDC was 68%–71% of the number recorded by the Guttmacher Institute through a national census of abortion providers (*8*,*9*,*16*).^§§§§§^In addition, whereas most reporting areas that send abortion data to CDC have laws requiring medical providers to submit a report for every abortion they perform to a central health agency, in New Jersey and DC medical providers submit this information voluntarily (*23*). As a result, the abortion numbers these areas report to CDC are likely incomplete.^¶¶¶¶¶^ Moreover, even in states that legally require medical providers to submit a report for all the abortions they perform, enforcement of this requirement varies, and as a consequence, numbers from multiple other reporting areas are likely incomplete as well.******

Second, because reporting requirements are established by the individual reporting areas, many states use reporting forms that differ from the technical standards and guidance CDC developed in collaboration with the National Association for Public Health Statistics and Information Systems. Consequently, many reporting areas do not collect all the information CDC compiles on the characteristics of women obtaining abortions (e.g., maternal age, race, and ethnicity) or do not report the data in a manner consistent with this guidance (e.g., gestational age). Although missing demographic information can reduce the extent to which the statistics in this report represent all women in the United States, five nationally representative surveys of women obtaining abortions in 1987, 1994–1995, 2001–2002, 2008, and 2014 (*17*–*20*,*22*) produced percentage distributions for most characteristics that are nearly identical to the percentage distributions reported by CDC. The exception is the percentage distribution of abortions by race/ethnicity. The percentage of abortions accounted for by non-Hispanic black women is higher and by Hispanic women is lower in this report than the percentages reported from a recent nationally representative survey of women obtaining abortions (*22*). Differences might be attributable to the fact that the number of states that report to CDC by race/ethnicity continues to be somewhat lower than for other demographic variables. Certain reporting areas that have not reported to CDC or have not reported cross-classified race/ethnicity data (e.g., California, Florida, and Illinois) have sufficiently large populations of minority women that the absence of data from these areas reduces the representativeness of CDC data.

In addition, certain areas collect gestational age on the basis of estimated date of conception or collect probable postfertilization age. Without medical guidance on how to report these data, the validity and reliability of gestational age for these reporting areas is uncertain.

Despite challenges in capturing medical abortions for reporting (*8*,*16*,*23*,*72*), a comparison of CDC data with mifepristone sales data^††††††^ suggests that CDC’s Abortion Surveillance System accurately describes the use of early medical abortion relative to other abortion methods in the United States (*73*). However, because of recent changes in clinical practice guidelines for the use of mifepristone and misoprostol through 9 completed weeks of gestation, CDC’s current definition of early medical abortion does not represent abortions performed through this method. Nonetheless, for 2015, of the medical abortions reported at ≤9 weeks, only 5.4% were performed at 9 weeks, and CDC continues to monitor these changes in clinical practice.

Third, abortion data are compiled and reported to CDC by the central health agency of the reporting area in which the abortion was performed rather than the reporting area in which the woman lived. Thus, the available population (*32*–*41*) and birth data (*42*), which are organized by the states in which women live, differ in certain cases from the population of women who undergo abortions in a given reporting area. This likely results in an overestimation of abortions for reporting areas in which a high percentage of abortions are obtained by out-of-state residents and an underestimation of abortions for states where residents frequently obtain abortions out of state. Limited abortion services, more stringent regulatory requirements for obtaining an abortion, or geographic proximity to services in another state might influence where women obtain abortion services. To examine these reporting biases, CDC attempts to categorize abortions by residence in addition to geographic occurrence. However, in 2015, CDC was unable to identify the reporting area, territory, or country of residence for 12.7% of reported abortions.

Finally, because reporting areas provide CDC with aggregate numbers, not individual-level data, and because available demographic information is limited by what reporting areas collect on their reporting forms, it is not possible to perform stratified analyses by additional demographic variables (e.g., socioeconomic status).

### Public Health Implications

Ongoing surveillance of legal induced abortion is important for several reasons. First, abortion surveillance is needed to guide and evaluate the success of programs aimed at preventing unintended pregnancies. Although pregnancy intentions can be difficult to assess (*74*–*79*), abortion surveillance provides an important measure of pregnancies that are unwanted. Second, routine abortion surveillance is needed to assess trends in clinical practice patterns over time. Information in this report on the number of abortions performed through different methods (e.g., medical or surgical) and at different gestational ages provides the denominator data that are necessary for analyses of the relative safety of abortion practices (*80*). Finally, information on the number of pregnancies ending in abortion is needed in conjunction with data on births and fetal losses to more accurately estimate the number of pregnancies in the United States and determine rates for various outcomes of public health importance (e.g., adolescent pregnancies) (*81*).

According to the most recent national estimates from 2010, 18% of all pregnancies in the United States end in induced abortion (*82*). Multiple factors influence the incidence of abortion, including access to health care services and contraception (*83*–*85*); the availability of abortion providers (*8*,*9*,*16*,*86*–*89*); state regulations, such as mandatory waiting periods (*66*), parental involvement laws (*90*), and legal restrictions on abortion providers (*91*,*92*); increasing acceptance of nonmarital childbearing (*93*,*94*); shifts in the race/ethnicity composition of the U.S. population (*95*,*96*); and changes in the economy and the resulting impact on fertility preferences and use of contraception (*97*,*98*). However, despite the multiple influences on abortion, because unintended pregnancy precedes nearly all cases of abortions,^§§§§§§^ efforts to help women avoid pregnancies that they do not desire might reduce the number of abortions (*83*–*85*).

Recent data indicate that the proportion of pregnancies in the United States that were unintended decreased from 51% in 2008 to 45% during 2011–2013, after a slight increase from 2001 to 2008 (*52*). Changing patterns of contraception use might have contributed to this decrease in unintended pregnancy. The use of the most effective forms of reversible contraception (i.e., intrauterine devices and hormonal implants) (*99*) has recently increased among all women (*100*–*103*), and the use of contraception overall appears to be increasing among adolescents (*104*). Of reported abortions in 2015, the majority were among women with a previous birth, and a substantial proportion occurred among women with a previous induced abortion, events that are also opportunities for contraception counseling. Contraception provision in the immediate postpartum and postabortion settings might increase access to these methods at a time when women are receiving health services. In addition, providing contraception for women at no cost can increase use of these methods and reduce abortion rates (*83*–*85*,*105*). Insufficient provider reimbursement and training, inadequate client-centered counseling, lack of youth-friendly services, and low client awareness of available contraceptive methods are also barriers to accessing contraception (*106*–*109*). Removing these barriers might help improve contraceptive use, potentially reducing the number of unintended pregnancies and the number of abortions performed in the United States.

## Footnotes

* Hereafter, all abortions in this report are considered to be legally induced unless stated to be illegally induced.

† Includes aspiration curettage, suction curettage, manual vacuum aspiration, menstrual extraction, sharp curettage, and dilation and evacuation procedures.

§ CDC collects information only on the estimated number of weeks (not days) of gestation and acknowledges the conventional use of completed weeks of gestation to describe pregnancy duration. CDC’s category ≤8 weeks’ gestation thus includes abortions up through 8 weeks and 6 days. This closely corresponds to the performance measure for medical abortion proposed by the American College of Obstetricians and Gynecologists (*27*).

¶ The cutoff of ≤12 weeks was selected on the basis of the implausibility of this procedure being performed at earlier gestational ages and on the basis of early research assessing the safety of intrauterine instillations starting at 13 weeks’ gestation (*29*,*30*).

** Excludes three reporting areas that did not report (California, Maryland, and New Hampshire), and one (Florida) that did not report by maternal residence.

†† States that did not report for ≥1 year since 1998 include Alaska (1998–2000), California (1998–2015), Louisiana (2005), Maryland (2007–2013), New Hampshire (1998–2013), Oklahoma (1998–1999), and West Virginia (2003–2004).

§§ Excludes California, Maryland, and New Hampshire.

¶¶ Comparisons do not include Wyoming, which reported <20 abortions.

*** Comparisons by residence status do not include California, Florida, Maryland, or New Hampshire. Because these areas either did not report or did not report abortions by maternal residence, numbers are available only from other reporting areas where their residents obtained abortions, and as a consequence meaningful statistics cannot be reported.

††† Comparisons do not include District of Columbia, which only reported abortions for area residents.

§§§ Arkansas and Texas reported probable postfertilization age. Two weeks were added to the probable postfertilization age to provide a corresponding measure to gestational age on the basis of the clinician’s estimate. Virginia reported clinician’s estimate of gestational age based on date of conception; no modifications were made to these data.

¶¶¶ Excludes Alabama, Arizona, California, Delaware, Florida, Georgia, Hawaii, Illinois, Kentucky, Louisiana, Maryland, Nevada, New Hampshire, New Mexico, Tennessee, Vermont, Wisconsin, and Wyoming.

**** Excludes Alabama, California, Connecticut, District of Columbia, Florida, Hawaii, Illinois, Kentucky, Louisiana, Maine, Maryland, Massachusetts, Mississippi, Nebraska, New Hampshire, New Mexico, New York State, Pennsylvania, Tennessee, Vermont, Wisconsin, and Wyoming.

†††† See Table 11 for list of reporting areas.

§§§§ Excludes Alaska, Arizona, California, Connecticut, Delaware, District of Columbia, Florida, Hawaii, Illinois, Iowa, Kentucky, Louisiana, Maine, Maryland, Massachusetts, Michigan, Mississippi, Nebraska, Nevada, New Hampshire, New Mexico, New York State, North Carolina, North Dakota, Oklahoma, Pennsylvania, Rhode Island, South Carolina, Vermont, Washington, Wisconsin, and Wyoming.

¶¶¶¶ Excludes Arizona, Arkansas, California, Connecticut, District of Columbia, Florida, Georgia, Louisiana, Maine, Maryland, Massachusetts, Nebraska, Nevada, New Hampshire, New York City, New York State, North Carolina, Ohio, Rhode Island, Vermont, Washington, and Wyoming.

***** Excludes California, Connecticut, Delaware, District of Columbia, Florida, Hawaii, Illinois, Maryland, Massachusetts, New Hampshire, New Mexico, New York State, North Carolina, Pennsylvania, Vermont, Wisconsin, and Wyoming.

††††† Excludes California, Connecticut, Delaware, District of Columbia, Florida, Georgia, Hawaii, Illinois, Maryland, Massachusetts, New Hampshire, New Mexico, New York State, North Carolina, Vermont, Wisconsin, and Wyoming.

§§§§§ In 2014, the most recent year for which the Guttmacher Institute has published data, abortions performed in California, Maryland, and New Hampshire accounted for 20% of the 926,200 abortions counted through the Guttmacher Institute’s national census of abortion providers (*16*).

¶¶¶¶¶ In 2014, the abortion numbers that CDC obtained from the District of Columbia and New Jersey were 48% and 54%, respectively, of the abortion numbers that the Guttmacher Institute obtained for these areas through their national census of abortion providers (*16*).

****** In 2014, the abortion numbers CDC obtained for Wyoming were <5% of the numbers obtained for this state by the Guttmacher Institute through their national census of abortion providers. CDC numbers for Hawaii were 57% of the Guttmacher Institute numbers. CDC numbers for Colorado, Connecticut, Nevada, New York (city and state combined), North Carolina, Oregon, Rhode Island, Tennessee, West Virginia, and Vermont were 74%–<90% of the Guttmacher Institute numbers. All other areas with legal reporting requirements that provided data to CDC obtained numbers that were at least 90% of the Guttmacher Institute numbers (*16*).

†††††† Because the sole distributor of mifepristone in the United States only sells this medication to licensed physicians, who must sign and return a prescriber’s agreement, sales data from this company are not limited by individual state reporting requirements or the difficulties of identifying smaller providers within the wider medical community.

§§§§§§ Recent estimates suggest that intended pregnancies account for <5% of all abortions (*31*).
