# *Erratum*: Vol. 67, No. SS-13

**DOI:** 10.15585/mmwr.mm6948a11

**Published:** 2020-12-04

In the Surveillance Summary “Abortion Surveillance — United States, 2015,” data on the number of known previous induced abortions for women having abortions in this reporting year were erroneously included for New York City. These estimates did not meet reporting standards and should have been excluded from this report. When corrected, among women with abortions in the reporting year, the proportion with no previous induced abortions increased, and the proportion with one or more previous induced abortions decreased.

On page 2, the second and third sentences of the second paragraph should have read “Women with one or more previous induced abortions accounted for **41.0%** of abortions, and women with no previous abortion accounted for **59.0%**. Women with three or more previous births accounted for 14.2% of abortions, and women with three or more previous abortions accounted for **6.5%** of abortions.”

On page 8, the last paragraph of the second column should have read “Data from the **38** areas that reported the number of previous abortions for women who obtained abortions in 2015 indicate that the majority (**59.0%**) had no previous abortions, **34.5%** had one or two previous abortions, and **6.5%** had three or more previous abortions (Table 17). Among the **34** reporting areas^†††††^ that provided data for the relevant years of comparison (2006 versus 2015, 2006 versus 2010, 2011 versus 2015, and 2014 versus 2015), the percentage of women who had no previous abortions increased **2%** (from **57.8% to 59.1%**), whereas there was a **4%** decrease for women who had one or two previous abortions, **and the percentage of women who had three or more previous abortions was unchanged (6.4%)** from 2006 to 2015. However, the percentage of women who had no previous abortions decreased 1% from 2006 to 2010 (from **57.8% to 57.4%**) and then increased **3%** from 2011 to 2015 (from **57.2% to 59.1%**). By contrast, the percentage of women who had three or more previous abortions increased **11.0%** from 2006 to 2010 (from **6.4% to 7.1%**) then decreased 9% from 2011 to 2015 (from **7.0% to 6.4%**). The percentage of women who had one or two previous abortions remained stable from 2006 to 2010 (**35.7% to 35.6%**) and then decreased 4% from 2011 to 2015 (from **35.8% to 34.4%**).” New York City should have been included in the ^†††††^ footnote, which lists reporting areas that were not included in these estimates.

In Table 17, the line for New York City should be deleted. For the total line, the numbers and percentages should have read **224,163 (59.0), 93,025 (24.5), 37,923 (10.0), 24,519 (6.5), 379,630 (99.4)**. The * footnote should have read “Data from **38** reporting areas; excludes **14** areas (California, Connecticut, District of Columbia, Florida, Hawaii, Illinois, Maryland, New Hampshire, New Mexico, **New York City**, New York State, North Carolina, Wisconsin, and Wyoming) that did not report, did not report by the number of previous induced abortions, or did not meet reporting standards.” The total in the ** footnote should have been **382,003**.

